# Evolution of Zygotic Linkage Disequilibrium in a Finite Local Population

**DOI:** 10.1371/journal.pone.0080538

**Published:** 2013-11-27

**Authors:** Xin-Sheng Hu

**Affiliations:** Department of Plant Sciences, University of Oxford, Oxford, United Kingdom; Centers for Disease Control and Prevention, United States of America

## Abstract

One crucial feature of zygotic linkage disequilibrium (LD) analysis is its direct use of diploid genotyping data, irrespective of the type of mating system. Previous theories from an evolutionary perspective mainly focus on gametic LD, but the equivalent development for zygotic LD is not available. Here I study the evolution of zygotic LD and the covariances between gametic and zygotic LDs or between distinct zygotic LDs in a finite local population under constant immigration from a continent population. I derive the analytical theory under genetic hitchhiking effects or in a neutral process. Results indicate that zygotic LDs (diploid level) are more informative than gametic LD (haploid level) in indicating the effects of different evolutionary forces. Zygotic LDs may be greater than or comparable to gametic LD under the epistatic selection process, but smaller than gametic LD under the non epistatic selection process. The covariances between gametic and zygotic LDs are strongly affected by the mating system, linkage distance, and genetic drift effects, but weakly affected by seed and pollen flow and natural selection. The covariances between different zygotic LDs are generally robust to the effects of gene flow, selection, and linkage distance, but sensitive to the effects of genetic drift and mating system. Consistent patterns exist for the covariances between the zygotic LDs for the two-locus genotypes with one common genotype at one locus or without any common genotype at each locus. The results highlight that zygotic LDs can be applied to detecting natural population history.

## Introduction

Zygotic linkage disequilibrium (LD) refers to the difference between the joint genotypic frequency at two loci and the product of genotypic frequencies at each locus [1], [2], [3], [4], [5], [6]. The concept itself is a purely statistical term, and can also be viewed as the covariance of genotypic frequencies, analogous to the covariance of allelic frequencies for the concept of gametic LD [7], [8], [9]. Its biological significance can be viewed when used for detecting the effects of evolutionary forces by comparing its empirical distribution with the predicted distribution once an evolutionary model is specified [9], [10]. The commonality between gametic and zygotic LDs lies in their utility for measuring non-random associations between loci. The crucial difference is that zygotic LD analysis does not require a random mating assumption since it directly uses diploid genotyping data. However, gametic LD calculation inferred from diploid genotypes needs this assumption since haplotypes must be priorly known. Such a difference is significant because the potential false-positive errors could be substantial in inferring haplotypes/linkage phases using the diploid genotyping data sampled from a natural population of a mixed mating system.

Previously relevant theories emphasize the joint frequency of double heterozygotes or double homozygotes in a neutral process, or the joint descent measures for a population with a mixed mating system [3], [4]. [11], [12], [13], [14], [15]. Zygotic LD is implicitly indicated from interpreting character associations in a partial inbreeding system [16], or from explaining an excess of the equilibrium genotypic frequencies at two independent loci in a mixed mating system [17], or from defining the covariance of heterozygosities [18] or the covariance of descent identities [19]. More recent studies concentrate on the statistical issues, including the procedure for testing zygotic LDs [5], [6], [20], [21], [22] and the potential application of zygotic LD to mapping quantitative trait loci (QTL) [23]. Unlike gametic LD that has received considerably theoretical studies from the evolutionary perspective [9], [10], [24], an equivalent theory for zygotic LD has not been fully developed. Although the evolutionary forces acting on the gametic LD may, in principle, also affect the zygotic LD, these effects and the resultant patterns have not been explicitly studied. This void motivates me to study how zygotic and other high-order LDs evolve under the effects of different evolutionary forces.

In flowering plants, three distinct processes in a life cycle are involved in changing zygotic LD and its relationship with gametic LD in a local population. One process is the asymmetric immigration through haploid pollen flow and diploid seed flow from a source population. Pollen flow directly generates gametic LD, but indirectly affects zygotic LD since each pollen grain only carries one gamete in fusion with ovules in the recipient population. Seed flow can generate both zygotic and gametic LDs since each seed carries two gametes into the recipient population simultaneously.

The second process influencing zygotic LD in plants is the mating system [25]. Selfing facilitates both gametic and zygotic LDs, even for the loci with a free recombination [17]; while random mating erodes both gametic and zygotic LDs. This effect can be unequal on zygotic and gametic LDs since zygotic LD might be more sensitive than gametic LD to the mating system.

The third process influencing zygotic LD in plants is selection in either the gametophyte or the sporophyte stage, or in both stages. Selection against heterozygote or epistatic selection at the sporophyte stage can directly change zygotic LD, but indirectly changes gametic LD [26], such as in natural hybrid zones [27], [28], [29], [30]. This is analogous to the conventional artificial selection that directly exerts effects on zygotic LD but indirectly on gametic LD in plant and animal breeding programs. Selection in the gametophyte stage directly changes gametic LD, but indirectly changes zygotic LD owing to the connection between the gametophyte and sporophyte stages in one life cycle. The natural overloading of pollen on the stigma of a flower implies pollen competition and the occurrence of natural selection [31], [32], [33]. Also, an excess of ovule abortion in many single embryo or polyembryony plants implies the occurrence of natural selection in ovules [34], [35]. Some genes can express at both the gametophyte and sporophyte stages [36], [37], [38], [39], [40], and might experience different extents of selection pressure. Selection can change both gametic and zygotic LDs among these genes. Thus, it is of both theoretical and practical significance to study how the above three distinct processes evolve zygotic LD.

Here I examine how different driving forces (mating system, genetic drift, migration, and natural selection) affect zygotic LD from the evolutionary perspective, complementary to the existing statistical issues. An island-continent model is considered, with an emphasis on the evolution of zygotic LD in the finite island population. I begin by presenting an exact model and use Monte Carlo (MC) simulations to evaluate the evolution of zygotic LD under different evolutionary forces in the population with a mixed mating system. I then derive analytical theories in two specific cases (genetic hitchhiking effects and a neutral process) under random mating, and validate the theories through MC simulations. Through the analytical and simulation results, I explore the evolutionary properties of zygotic LDs and discuss their potential utility.

## Results

### Exact Model

Consider an island population, with constant immigration rates of pollen (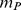 ) and seeds (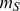) per generation from a continent population. The continent population is assumed to be sufficiently large in size and stable in genetic composition. Migration from the island to continent population is neglected, and mutation effects are excluded. At the gametophyte stage, pollen and ovules are subject to natural selection before they are combined to produce seeds. The plant life cycle follows a sequence of events: pollen and ovules generation, pollen flow, selection at the gametophyte stage, mixed mating with a selfing rate α (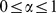), seed flow, selection at the sporophyte stage, and genetic drift. Selection strength may be either strong or weak; or epistatic selection is allowed in either the gametophyte or the sporophyte stage, or in both. The same mating system is assumed in the island and continent populations.

Consider two diallelic nuclear loci, with alleles 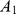
and 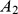 at locus *A*, alleles 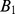 and 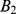 at locus *B*, and a recombination rate 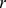 between the two loci. These alleles may refer to single nucleotide polymorphic (SNP) markers since tri- or tetra-allelic SNP sites are infrequent in natural populations. In the island population, let 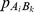
(*i*, *k* = 1, 2) and 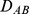
be the frequency of gamete 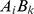 and the gametic LD in current adults, respectively; and 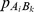
can be expressed as 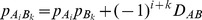. For the random mating part, the frequency of gamete 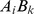 in pollen or ovules (the next generation) produced by the current adults can be expressed as 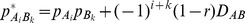 [41]. Similarly, let 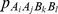, 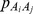, and 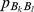 be the frequencies of genotypes 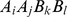, 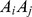 and 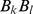
(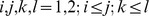) in the current adult population, respectively. Let 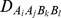 be the zygotic LD between genotypes 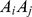 at locus *A* and 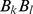
at locus *B* for two-locus genotype 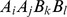, i.e. 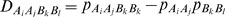. There are eight zygotic LDs in total, but only four of them are independent since the following constraints hold: 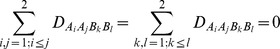. This leads to 
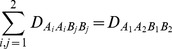
 and 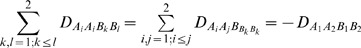 [5], [6].

In the continent population, let 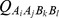,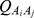, and 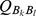 be the frequencies of genotypes 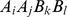, 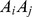 and 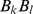
(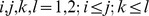), respectively. Let 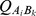,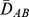, and 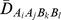
be the frequency of gamete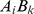, the gametic LD, and the zygotic LD in the current adults, respectively. Similar constraints for zygotic LDs to the case in the island population hold as well. All zygotic and gametic LDs are assumed to be constant in the continent population.

Let 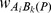 and 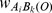 be the fitness of gamete 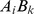 in pollen and ovules, respectively. The average fitness in pollen and ovules, denoted by 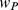 and 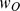, respectively, can be expressed as 
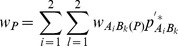
and 
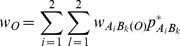
where 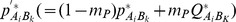 is the gametic frequency after pollen flow. The gametic frequencies in ovules remain unaltered since ovules do not move after pollen flow. Let 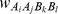 be the fitness for 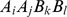 (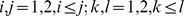). The average fitness in the sporophyte stage, *w*, can be calculated by 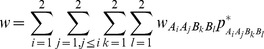
where 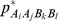 is the genotypic frequency after seed flow. Following the plant life cycle, the genotypic frequency after selection in the sporophyte stage, denoted by 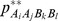 (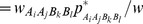 ) for 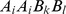
(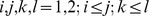), is derived in Appendix S1.

After genetic drift, the numbers of distinct genotypes follow a multinomial distribution. Here, the genetic sampling of *N* breeding individuals (an effective population size) is analogous in technique to but different in biological meaning from the statistical sampling of *N* individuals [9]. Allelic and genotypic frequencies fluctuate but eventually reach steady-state distributions under the joint effects of migration, selection, and genetic drift. Gametic and zygotic LDs can eventually reach steady-state distributions as well. Since the probability density functions (pdf) of gametic and zygotic LDs are difficult to derive, their expectations can be indirectly evaluated through multiple independent simulations.

Genetic drift at each generation can cause the associations between gametic and zygotic LDs or between different zygotic LDs due to their sharing of some alleles or genotypes. There are four types of covariances between gametic and zygotic LDs, 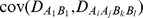 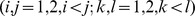, and six types of covariances between distinct zygotic LDs. Note that other high-order LDs, such as trigenic and composite LDs [9], are not examined here although they can be calculated with more complicated analyses. Fisher’s delta method is used to approximate the covariances between gametic and zygotic LDs or between different zygotic LDs (high-order LDs) ([9], p118), [42].

For example, the covariance between 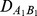
and 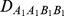 or between 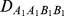
and 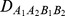 is derived as:
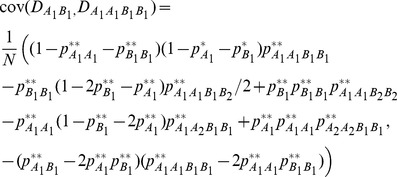
(1)

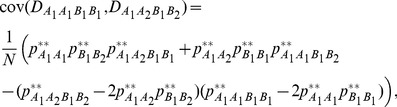
(2)where 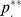
is the allelic or genotypic frequency after selection in the sporophyte stage but before genetic drift. Other high-order covariances can be derived in a similar way. These covariances are needed for calculating the expectations of zygotic LDs described in the section of Analytical Theory [10]. Note that the above covariances produced by genetic drift are conditional on the allelic and genotypic frequencies 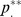
before genetic drift, i.e. the expectation on the basis of genetic drift (see 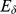
in Appendix S4). These high-order covariances can reach steady-state distributions, and their means, e.g.,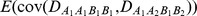, can be calculated in theory according to their joint probability density distribution (the expectation *E* is based on the pdf,
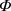
_,_ described in the section of Analytical Theory). Similarly, multiple independent simulations can be used to evaluate the expectations of these high-order LDs.

Note that the above general model can reduce to specific models with different numbers of evolutionary forces (e.g., the model with a single evolutionary force). Also, I only concentrate on the covariances between allelic frequencies, or between genotypic frequencies, or between gametic and zygotic LDs, or between different zygotic LDs. The expectations of their normalized values, like the square of normalized gametic LD, 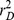 [8] or Lewontin’s 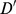 [43], are difficult to derive under genetic drift effects [44], and hence are not explored further.

### Monte Carlo Simulations

MC simulations are used to examine how different evolutionary forces change zygotic LDs and other types of covariances in the plant species of a mixed mating system. Suppose that the island population initially has the same genetic composition as the continent population. For simplicity, notation for the alleles and subscripts in the above exact model is changed as *A* for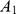, *a* for 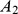, *B* for 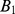, and *b* for 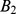. Simulations are conducted according to the plant life cycle. Given a set of parameters, including the genotypic frequencies in migrants and in the initial island population, the selection coefficients and the effective population size, the genotypic frequencies before genetic drift are calculated from Eqs. (A1) ∼ (A5) in Appendix S1. For the genetic drift, random samples are generated using the genotypic frequencies that follow a multinomial distribution. Random numbers with uniform distribution within (0, 1) for sampling purpose are generated using the routine of Press et al. ([45], pp. 210–211). Ten thousand independent simulation runs are conducted for each case. The replicates are used to estimate means and standard deviations of zygotic LDs and other covariances.

**Figure 1:**
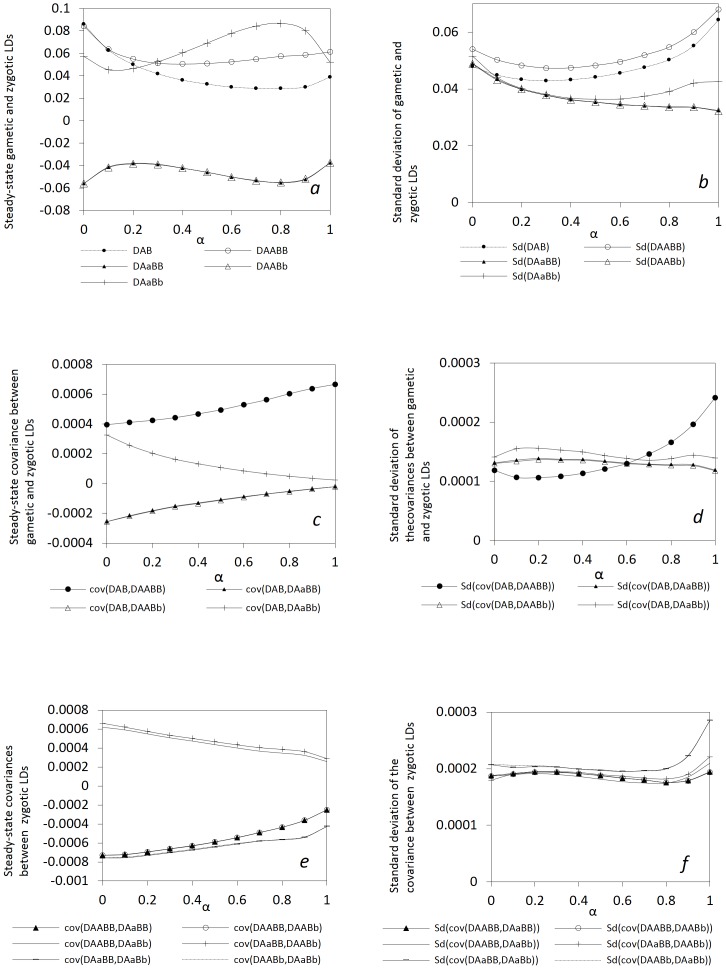
Effects of selfing on the steady-state gametic and zygotic LDs and other types of covariances. Average steady-state gametic and zygotic LDs (a) and their standard deviations (b); average steady-state covariances between gametic and zygotic LDs (c) and their standard deviations (d); and average steady-state covariances between distinct zygotic LDs (e) and their standard deviations (f). Results are obtained from 10000 independent simulation runs. Parameter settings are the recombination rate = 5%, the immigration rate of pollen *m_P_* = 0.08 and seeds *m_S_* = 0.04, the effective population size = 50, the fitness in the gametophyte stage (pollen and ovules) *w_AB_* = 1, *w_Ab_* = 0.98, *w_aB_* = 0.98, *w_ab_* = 0.96, and the fitness in the sporophyte stage *w_AABB_* = 1, *w_AABb_* = *w_AaBB_* = 0.98, *w_AAbb_* = *w_AaBb_* = *w_aaBB_* = 0.96, *w_Aabb_* = *w_aaBb_* = 0.94, and *w_aabb_* = 0.92. The genotypic frequencies in the continent and initial island populations are 0.1225 for *AABB*, 0.105 for *AABb*, 0.0225 for *AAbb*, 0.105 for *AaBB*, 0.245 for *AB/ab*, 0.045 for *ab/aB*, 0.105 for *Aabb*, 0.0225 for *aaBB*, 0.105 for *aaBb*, and 0.1225 for *aabb*.

**Mating System.** To examine the effects of mating system, I fix all other parameters except the selfing rate 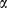. Here, gametic and zygotic LDs are not further decomposed into the components of identity (inbreeding in recent ancestry) and non-identity disequilibria [3], [4], and hence the interaction between selfing and genetic drift is unnecessarily specified. Simulations confirm that gametic and zygotic LDs and other covariances can reach steady-state distributions. Note that the parameter settings in all numerical examples are arbitrary as long as these parameters are biologically meaningful. Figure 1a (a coupling linkage phase, *D_AB_*>0) shows that the steady-state gametic and zygotic LDs have different patterns although they exhibit non monotonic trends with the selfing rate. Their standard deviations also exhibit non monotonic trends with 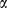 (Figure 1b). Thus, gametic and zygotic LDs are not a linear function of 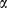, similar to the result in a cytonuclear system [46]. An overlap between the steady-state *E*(*D_AaBB_* ) and *E*(*D_AABb_*) is expected when the two loci initially have the same settings in selection coefficients and genotypic frequencies. There are the same patterns between the steady-state *E*(*cov*(*D_AB_*, *D_AABb_*)) and *E*(*cov*(*D_AB_*, *D_AaBB_*)), or between the steady-state *E*(*cov*(*D_AABB_*, *D_AaBB_*)) and *E*(*cov*(*D_AABB_*, *D_AaBB_*)), or between the steady-state *E*(*cov*(*D_AaBB_*, *D_AaBb_*)) and *E*(*cov*(*D_AABb_*, *D_AaBb_*)) in this example. Selfing increases homozygosity but reduces heterozygosity, resulting in different patterns among gametic and zygotic LDs. The steady-state *E*(*D_AB_*) may be smaller than the steady-state expectations of some zygotic LDs in a predominant selfing species (e.g., *E*(*D_AABB_* ) in Figure 1a).

The steady-state covariances between gametic and zygotic LDs (Figure 1c) or between distinct zygotic LDs (Figure 1e) exhibit a monotonic pattern with 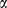. Selfing facilitates the covariances between gametic and zygotic LDs for the genotypes with homozygotes at one locus (the steady-state *E*(*cov*(*D_AB_*, *D_AABb_*)) and *E*(*cov*(*D_AB_*, *D_AaBB_*))) or at two loci (the steady-state *E*(*cov*(*D_AB_*, *D_AABB_*)), but reduces the steady-state *E*(*cov*(*D_AB_*, *D_AaBb_*)). Their standard deviations exhibit different patterns with the selfing rate (Figure 1d). Selfing also facilitates the covariances of zygotic LDs between the genotypes sharing one homozygote (the steady-state *E*(*cov*(*D_AABB_*, *D_AaBB_*)) and *E*(*cov*(*D_AABB_*, *D_AABb_*))) or sharing one heterozygote (the steady-state *E*(*cov*(*D_AaBB_*, *D_AaBb_*)) and *E*(*cov*(*D_AABb_*, *D_AaBb_*))), but reduces the covariances of zygotic LDs between the genotypes without any common genotypes (the steady-state *E*(*cov*(*D_AABB_*, *D_AaBb_*)) and *E*(*cov*(*D_AaBB_*, *D_AABb_*))). The standard deviations for these high-order LDs are stable with the selfing rate except their slight increases at the complete selfing (no effects from pollen flow at 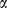 = 1; Figure 1f).

The steady-state *E*( *D_AB_* ) and *E*(*D_AABB_*) exhibit different patterns with the selfing rate between the repulsion (*D_AB_*<0) and coupling (*D_AB_*>0 ) linkage phases although they have similar patterns in each linkage phase. The steady-state *E*(*D_AaBb_*) and *E*(*D_AaBB_*) (or *E*(*D_AABb_*)) display similar patterns with
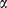
in each linkage-phase. Patterns are also similar between two linkage-phase cases for the steady-state *E*(*cov*(*D_AB_*, *D_AABB_*)), but not for other three covariances between gametic and zygotic LDs. Selfing reduces the absolute steady-state *E*(cov(*D_AB_*, *D_AaBb_*)) in each linkage phase. All covariances between different zygotic LDs have the same responding patterns to the selfing rate
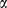
in each linkage phase (data not shown here).

The above examples indicate that plants with distinct mating systems have different zygotic LDs and other covariances in a local population even under the same impacts of immigration. Both zygotic and gametic LDs are sensitive to the pattern of mating system. Predominant outcrossing species have weaker covariances between zygotic and gametic LDs, but stronger covariances between distinct zygotic LDs than do the predominant selfing species.

**Figure 2:**
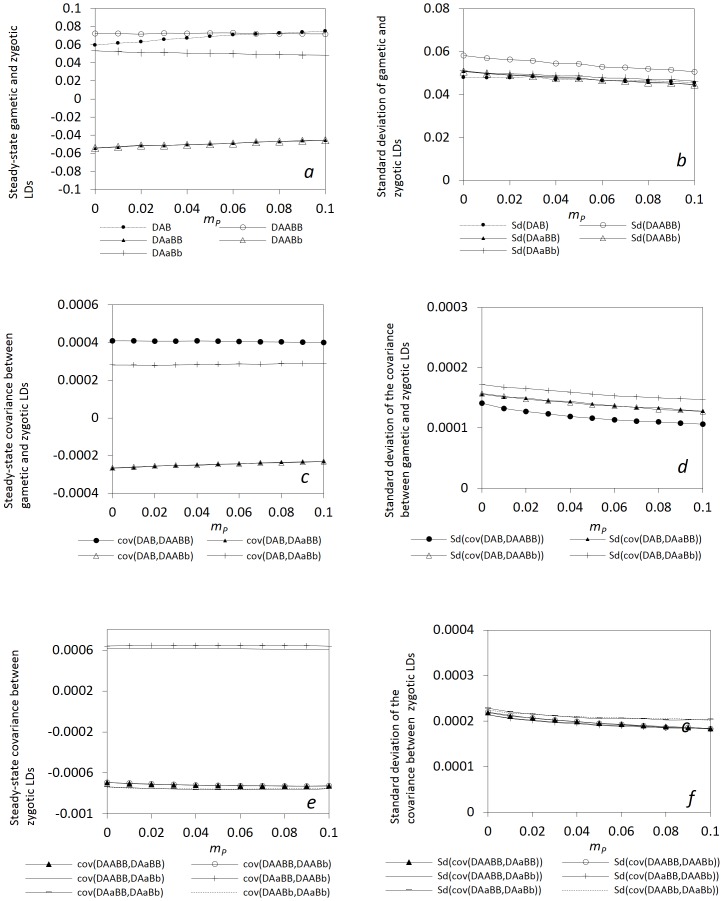
Effects of pollen flow on the steady-state gametic and zygotic LDs and other types of covariances. Average steady-state gametic and zygotic LDs (a) and their standard deviations (b); average steady-state covariances between gametic and zygotic LDs (c) and their standard deviations (d); and average steady-state covariances between distinct zygotic LDs (e) and their standard deviations (f). Results are obtained from 10000 independent simulation runs. Parameter settings are the selfing rate  = 5%, the recombination rate = 5%, the effective population size = 50, the immigration rate of seeds *m_S_* = 0.04, and the fitness in the gametophyte stage (pollen and ovules) *w_AB_* = 1, *w_Ab_* = 0.98, *w_aB_* = 0.98, *w_ab_* = 0.96, and the fitness in the sporophyte stage *w_AABB_* = 1, *w_AABb_* = *w_AaBB_* = 0.98, *w_AAbb_* = *w_AaBb_* = *w_aaBB_* = 0.96, *w_Aabb_* = *w_aaBb_* = 0.94, and *w_aabb_* = 0.92. The genotypic frequencies in the continent and initial island populations are 0.1225 for *AABB*, 0.105 for *AABb*, 0.0225 for *AAbb*, 0.105 for *AaBB*, 0.245 for *AB/ab*, 0.045 for *ab/aB*, 0.105 for *Aabb*, 0.0225 for *aaBB*, 0.105 for *aaBb*, and 0.1225 for *aabb*.

**Seed and Pollen Flow.** To examine the effects of pollen (or seed) flow, I fix all other parameters except the migration rate of pollen (or seeds). Figure 2a shows that the steady-state *E*(*D_AB_*) slightly increases with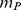 in a coupling linkage phase (*D_AB_*>0). The steady-state *E*(*D_AABB_* ) and *E*(*D_AaBb_* ) slightly decrease with 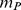, while the steady-state *E*(*D_AaBB_*) and *E*(*D_AABb_*) (negative) slightly increase with 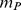
(Figure 2a). The steady-state *E*(*cov*(*D_AB_*, *D_AABb_*)), *E*(*cov*(*D_AB_*, *D_AaBB_*)), and *E*(*cov*(*D_AB_*, *D_AaBb_*)) slightly increase with 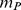 while the steady-state *E*(*cov*(*D_AB_*, *D_AABB_*)) decreases with 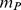
(Figure 2c). All covariances between different zygotic LDs slightly decrease with
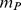
(Figure 2e). All standard deviations slightly decrease with 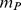
(Figures 2b, d, and f).

**Figure 3:**
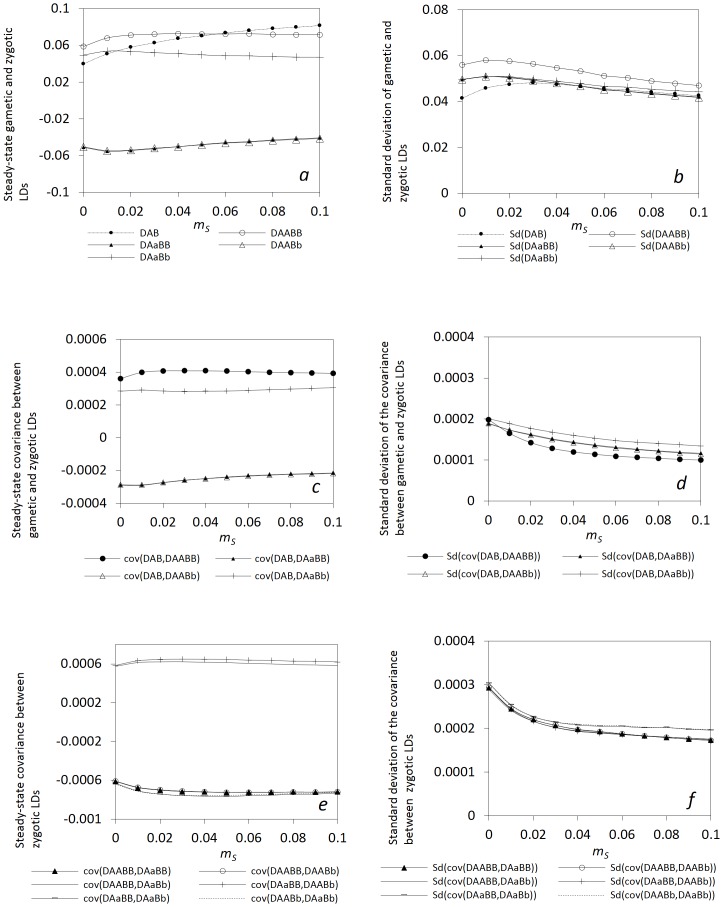
Effects of seed flow on the steady-state gametic and zygotic LDs and other types of covariances. Average steady-state gametic and zygotic LDs (a) and their standard deviations (b); average steady-state covariances between gametic and zygotic LDs (c) and their standard deviations (d); and average steady-state covariances between distinct zygotic LDs (e) and their standard deviations (f). Results are obtained from 10000 independent simulation runs. Parameter settings are the selfing rate  = 5%, the effective population size = 50, the immigration rate of pollen *m_P_* = 0.04, and the fitness in the gametophyte stage (pollen and ovules) *w_AB_* = 1, *w_Ab_* = 0.98, *w_aB_* = 0.98, *w_ab_* = 0.96, and the fitness in the sporophyte stage *w_AABB_* = 1, *w_AABb_* = *w_AaBB_* = 0.98, *w_AAbb_* = *w_AaBb_* = *w_aaBB_* = 0.96, *w_Aabb_* = *w_aaBb_* = 0.94, and *w_aabb_* = 0.92. The genotypic frequencies in the continent and initial island populations are 0.1225 for *AABB*, 0.105 for *AABb*, 0.0225 for *AAbb*, 0.105 for *AaBB*, 0.245 for *AB/ab*, 0.045 for *ab/aB*, 0.105 for *Aabb*, 0.0225 for *aaBB*, 0.105 for *aaBb*, and 0.1225 for *aabb*.

Seed flow has greater effects than pollen flow on zygotic LDs and other covariances (Figure 3; the same parameter settings as in Figure 2 except the different migration rates of seeds and pollen). The steady-state *E*(*D_AB_*) changes faster with
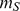
than any steady-state zygotic LDs. Generally, the steady-state zygotic LDs and the covariances between different zygotic LDs or between gametic and zygotic LDs do not monotonically change with 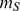. The steady-state *E*( *D_AABB_*) and *E*(*D_AaBb_*) slightly increase as
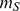
approaches the value of selection coefficient, and then slightly decrease afterwards (Figure 3a). Similar patterns exist for the change of the steady-state *E*(*cov*(*D_AB_*, *D_AABB_*)), *E*(*cov*(*D_AB_*, *D_AaBb_*)), *E*(*cov*(*D_AABB_*, *D_AaBb_*)), and *E*(*cov*(*D_AaBB_*, *D_AABb_*)) with 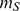
(Figures 3c and e). To the contrary, the steady-state *E*(*D_AaBB_*) and *E*(*D_AABb_*) slightly decrease as 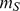
approaches the value of selection coefficient, and then slightly increase afterwards. Similar patterns exist for the change of the steady-state *E*(*cov*(*D_AB_*, *D_AaBB_*)), *E*(*cov*(*D_AB_*, *D_AABb_*)), *E*(*cov*(*D_AABB_*, *D_AaBB_*)), *E*(*cov*(*D_AABB_*, *D_AABb_*)), *E*(*cov*(*D_AaBB_*, *D_AaBb_*)), and *E*(*cov*(*D_AABb_*, *D_AaBb_*)) (Figures 3b and c). The same pattern exists for the covariances between zygotic LDs for the genotypes with a common genotype at one locus, or for the genotypes without any common genotype at each locus. All standard deviations gradually decrease with 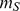 (Figures 3b, d, and f).

These examples indicate that a local plant population generally exhibits robust responses to the impacts of immigration of pollen and seeds in terms of zygotic LDs, or the covariances between gametic and zygotic LDs, or the covariances between distinct zygotic LDs. Seed and pollen flow have small effects on high-order LDs in a local population.

**Figure 4:**
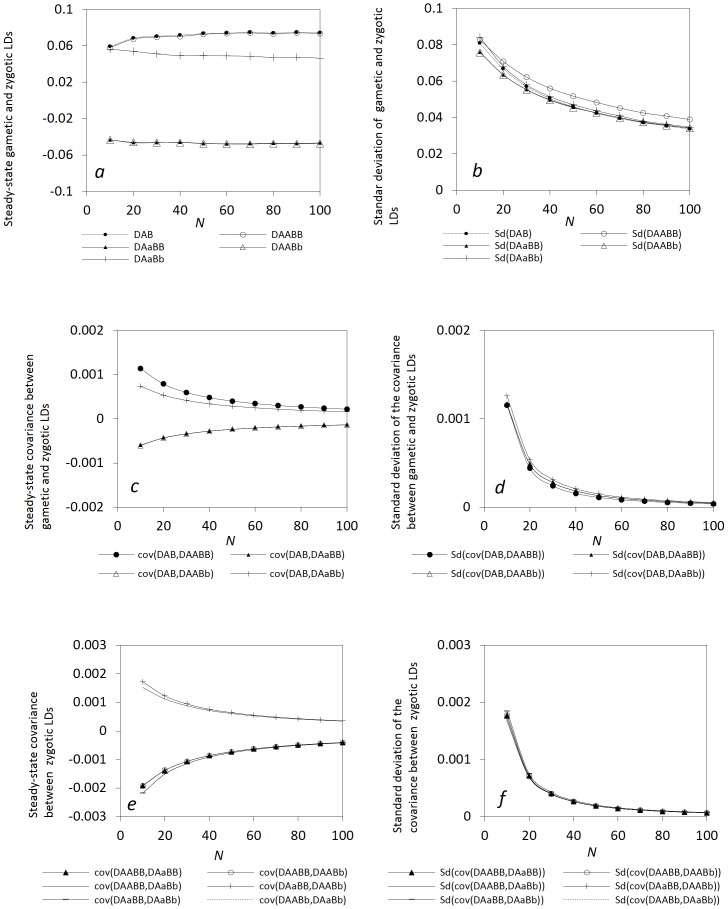
Genetic drift effects on the steady-state gametic and zygotic LDs and other types of covariances. Average steady-state gametic and zygotic LDs (a) and their standard deviations (b); average steady-state covariances between gametic and zygotic LDs (c) and their standard deviations (d); and average steady-state covariances between distinct zygotic LDs (e) and their standard deviations (f). Results are obtained from 10000 independent simulation runs. Parameter settings are the selfing rate  = 5%, the immigration rate of seeds *m_S_* = 0.04 and pollen *m_P_* = 0.08, and the fitness in the gametophyte stage (pollen and ovules) *w_AB_* = 1, *w_Ab_* = 0.98, *w_aB_* = 0.98, *w_ab_* = 0.96, and the fitness in the sporophyte stage *w_AABB_* = 1, *w_AABb_* = *w_AaBB_* = 0.98, *w_AAbb_* = *w_AaBb_* = *w_aaBB_* = 0.96, *w_Aabb_* = *w_aaBb_* = 0.94, and *w_aabb_* = 0.92. The genotypic frequencies in the continent and initial island populations are 0.1225 for *AABB*, 0.105 for *AABb*, 0.0225 for *AAbb*, 0.105 for *AaBB*, 0.245 for *AB/ab*, 0.045 for *ab/aB*, 0.105 for *Aabb*, 0.0225 for *aaBB*, 0.105 for *aaBb*, and 0.1225 for *aabb*.

**Genetic Drift.** To examine the effects of genetic drift, I fix all other parameters except the effective population size (*N*). Figure 4 shows the results for a predominant outcrossing species (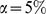). The steady-state *E*(*D_AB_* ) and *E*(*D_AABB_*) slightly increase with *N*. The steady-state *E*(*D_AaBB_*), *E*(*D_AABb_*), and *E*(*D_AaBb_*) (genotypes with heterozygote at one locus or two loci) slightly decrease as the effective population size increases (Figure 4a). The steady-state *E*(*cov*(*D_AB_*, *D_AABB_*)) and *E*(*cov*(*D_AB_*, *D_AaBb_*)) gradually reduce to zero as *N* increases. To the contrary, the steady-state *E*(*cov*(*D_AB_*, *D_AABb_*)) and *E*(*cov*(*D_AB_*, *D_AaBB_*)) gradually increase to zero as *N* increases (Figure 4c). The steady-state *E*(*cov*(*D_AABB_*, *D_AaBb_*)) and *E*(*cov*(*D_AaBB_*, *D_AABb_*)) gradually reduce to zero with *N*, while other steady-state *E*(*cov*(*D_AABB_*, *D_AaBB_*)), *E*( *cov*(*D_AABB_*, *D_AABb_*)), *E*(*cov*(*D_AaBB_*, *D_AaBb_*)), and *E*(*cov*(*D_AABb_*, *D_AaBb_*)) gradually increase to zero with *N* (Figure 4e). It is clear that covariances between gametic and zygotic LDs or between different zygotic LDs are more sensitive than gametic and zygotic LDs to the genetic drift effects. All standard deviations gradually decrease with *N* (Figures 4b, d, and f).

The examples indicate that a small local population can affect zygotic LDs, and has large effects on the covariances between gametic and zygotic LDs or between distinct zygotic LDs. These high-order covariances are more informative than gametic LD in signaling the effects of population demographic dynamics.

**Selection.** To assess the effects of linear-additive selection, I examine three selection schemes: gametic selection only, zygotic selection only, and both gametic and zygotic selection. Table 1 shows a comparison in the steady-state zygotic and gametic LDs and other types of covariances. The steady-state *E*(*D_AB_*) slightly decreases while the absolute steady-state zygotic LDs and other types of covariances increase from the case of gametic selection only to the case of zygotic selection only, and to the case of joint selection. The examples indicate that cumulative selection can enhance zygotic LDs and other covariances in the linear additive-viability model (Table 1).

**Table 1:** Effects of selection in the gametophyte stage, the sporophyte stage, and in both stages on the steady-state gametic and zygotic LDs and other high-order covariances*

	Gametic selection	Zygotic selection	Gametic and zygotic selection
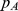	0.5780± 0.1221	0.5893±0. 1217	0.6521±0.1135
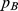	0.5822±0.1224	0.5919±0.1209	0.6559±0.1131
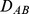	0.0755±0.0497	0.0746±0.0495	0.0730±0.0463
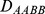	0.0619±0.0495	0.0629±0.0499	0.0722±0.0519
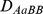	–0.0338±0.0427	–0.0355±0.0433	–0.0468±0.0456
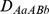	–0.0342±0.0428	–0.0358±0.0431	–0.0471±0.0456
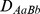	0.0437±0.0468	0.0438±0.0467	0.0492±0.0471
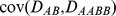	3.619×10^−4^±1.101×10^−4^	3.679×10^−4^±1.107×10^−4^	4.035×10^−4^±1.099×10^−4^
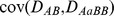	–1.813×10^−4^±1.341×10^−4^	–1.887×10^−4^±1.350×10^−4^	–2.348×10^−4^±1.329×10^−4^
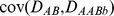	–1.840×10^−4^±1.329×10^−4^	–1.904×10^−4^±1.338×10^−4^	–2.368×10^−4^±1.313×10^−4^
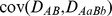	2.691×10^−4^±1.615×10^−4^	2.689×10^−4^±1.616×10^−4^	2.880×10^−4^±1.508×10^−4^
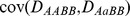	–6.525×10^−4^±2.086×10^−4^	–6.653×10^−4^±2.064×10^−4^	–7.266×10^−4^±1.877×10^−4^
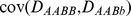	–6.550×10^−4^±2.088×10^−4^	–6.665×10^−4^±2.056×10^−4^	–7.280×10^−4^±1.874×10^−4^
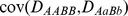	5.107×10^−4^±2.043×10^−4^	5.256×10^−4^±2.025×10^−4^	6.088×10^−4^±1.870×10^−4^
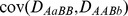	5.500×10^−4^±2.083×10^−4^	5.636×10^−4^±2.059×10^−4^	6.428×10^−4^±1.855×10^−4^
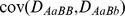	–6.816×10^−4^±2.344×10^−4^	–6.945×10^−4^±2.297×10^−4^	–7.631×10^−4^±2.035×10^−4^
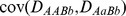	–6.723×10^−4^±2.338×10^−4^	–6.892×10^−4^±2.316×10^−4^	–7.562×10^−4^±2.051×10^−4^

* Three selection schemes are: *w_AB_* = 1,*w_Ab_* = 0.98, *w_aB_* = 0.98, and *w_ab_* = 0.96 for gametic selection only; *w_AABB_* = 1, *w_AABb_* = *w_AaBB_* = 0.98, *w_AAbb_* = *w_AaBb_* = *w_aaBB_* = 0.96, *w_Aabb_* = *w_aaBb_* = 0.94, and *w_aabb_* = 0.92 for zygotic selection only; and *w_AB_* = 1,*w_Ab_* = 0.98, *w_aB_* = 0.98, *w_ab_* = 0.96, *w_AABB_* = 1, *w_AABb_* = *w_AaBB_* = 0.98, *w_AAbb_* = *w_AaBb_* = *w_aaBB_* = 0.96, *w_Aabb_* = *w_aaBb_* = 0.94, and *w_aabb_* = 0.92 for both gametic and zygotic selection. Other parameter settings are the recombination rate = 5%, the immigration rate of pollen *m_P_* = 0.08 and seeds *m_S_* = 0.04, and the effective population size = 50. The genotypic frequencies in the continent and initial island populations are 0.1225 for *AABB*, 0.105 for *AABb*, 0.0225 for *AAbb*, 0.105 for *AaBB*, 0.245 for *AB/ab*, 0.045 for *ab/aB*, 0.105 for *Aabb*, 0.0225 for *aaBB*, 0.105 for *aaBb*, and 0.1225 for *aabb*. The steady-state results (mean ± *S_d_*) are obtained from 10000 independent simulation runs.

To assess the effects of epistatic selection, I use Dobzhansky-Muller’s incompatibility model [27], [28], [47] as an example to demonstrate how epistatic selection in the sporophyte stage affects gametic and zygotic LDs. Three cases with different extents of epistatic selection are examined. Selection in the gametophyte stage is excluded in each case. In Case I, the genotypic fitness is set as *w_AABB_* = *w_aabb_* = 1, *w_AaBB_* = *w_aaBb_* = 0.99, *w_AABb_* = *w_Aabb_* = 0.99, *w_aaBB_* = 0.98, *w_AAbb_* = 0.98, and *w_AaBb_* = 0.98. In Case II, the genotypic fitness is set as *w_AABB_* = *w_aabb_* = 1, *w_AaBB_* = *w_aaBb_* = 0.99, *w_AABb_* = *w_Aabb_* = 0.5, *w_aaBB_* = 0.98, *w_AAbb_* = 0.5, and *w_AaBb_* = 0.5. In Case III, the genotypic fitness is set as *w_AABB_* = *w_aabb_* = 1, *w_AaBB_* = *w_aaBb_* = 0.99, *w_AABb_* = *w_Aabb_* = 0.1, *w_aaBB_* = 0.98, *w_AAbb_* = 0.1, and *w_AaBb_* = 0.1. These three cases are the same as matrices (13), (14), and (15) of Gavrilets [48], respectively. In these settings, alleles *A* and *b* have a progressively negative interaction on fitness (incompatible background interactions) from Cases I to III.

Results indicate that epistatic selection can change the relative gametic and zygotic LDs (Table 2). The steady-state frequency of allele *B* increases while the steady-state frequency of allele *A* decreases from Cases I to III. The steady-state *E*(*D_AaBB_*) and absolute steady-state *E*(*D_AaBb_*) become greater than the steady-state *E*(*D_AB_* ) in Case III. The steady-state *E*(*D_AB_*), *E*(*D_AABB_*), and *E*(*D_AaBb_*) decrease while the steady-state *E*(*D_AaBB_*) and *E*(*D_AABb_*) increase from Cases I to III. Epistatic selection also changes the covariances between gametic and zygotic LDs or between different zygotic LDs. The steady-state *E*(*cov*(*D_AB_*, *D_AABB_*)*)*, *E*(*cov*(*D_AB_*, *D_AABb_*)), and *E*(*cov*(*D_AB_*, *D_AaBb_*)) decrease while the steady-state *E*(*cov*(*D_AB_*, *D_AaBB_*)) increases from Cases I to III. The steady-state *E*(*cov*(*D_AABB_*, *D_AABb_*)), *E*(*cov*(*D_AABB_*, *D_AaBb_*)), and *E*(*cov*(*D_AaBB_*, *D_AABb_*)) decrease while the steady-state *E*(*cov*(*D_AABB_*, *D_AaBB_*)), *E*(*cov*(*D_AaBB_*, *D_AaBb_*)), and *E*(*cov*(*D_AABb_*, *D_AaBb_*)) increase from Cases I to III.

**Table 2:** Effects of Dobzhansky-type epistatic selection on the steady-state gametic and zygotic LDs and other high-order covariances*

	Case I	Case II	Case III
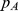	0.5068± 0.1280	0.3090±0. 2127	0.2673±0.2343
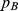	0.5078±0.1292	0.7573±0.1937	0.8170±0.2009
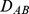	0.0788±0.0510	0.0329±0.0218	0.0165±0.0113
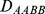	0.0533±0.0462	0.0219±0.0236	0.0100±0.0112
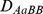	–0.0235±0.0394	0.0257±0.0337	0.0310±0.0248
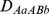	–0.0231±0.0397	–0.0172±0.0205	–0.0079±0.0095
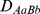	0.0436±0.0474	–0.0120±0.0312	–0.0245±0.0215
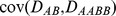	3.217×10^−4^±1.149×10^−4^	1.368×10^−4^±1.195×10^−4^	0.556×10^−4^±7.466×10^−4^
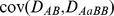	–1.384×10^−4^±1.353×10^−4^	0.6650×10^−4^±1.158×10^−4^	8.250×10^−4^±6.508×10^−4^
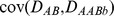	–1.399×10^−4^±1.341×10^−4^	–0.9688×10^−4^±0.8927×10^−4^	–0.377×10^−4^±0.467×10^−4^
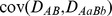	2.692×10^−4^±1.642×10^−4^	0.1426×10^−4^±1.194×10^−4^	–0.499×10^−4^±0.505×10^−4^
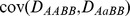	–5.511×10^−4^±2.298×10^−4^	–0.928×10^−4^±1.275×10^−4^	0.1252×10^−4^±0.537×10^−4^
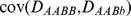	–5.518×10^−4^±2.289×10^−4^	–1.987×10^−4^±1.753×10^−4^	–0.6276×10^−4^±0.823×10^−4^
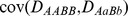	3.942×10^−4^±2.105×10^−4^	0.8561×10^−4^±1.252×10^−4^	–0.092×10^−4^±0.486×10^−4^
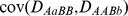	4.386×10^−4^±2.212×10^−4^	0.8903×10^−4^±1.284×10^−4^	–0.091×10^−4^±0.489×10^−4^
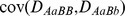	–5.628×10^−4^±2.536×10^−4^	–5.495×10^−4^±2.362×10^−4^	–2.307×10^−4^±1.544×10^−4^
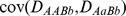	–5.611×10^−4^±2.513×10^−4^	–0.7594×10^−4^±1.251×10^−4^	0.128×10^−4^±0.4867×10^−4^

* Three selection schemes are: *w_AABB_* = *w_aabb_* = 1, *w_AaBB_* = *w_aaBb_* = 0.99, *w_AABb_* = *w_Aabb_* = 0.99, *w_aaBB_* = 0.98, *w_AAbb_* = 0.98, and *w_AaBb_* = 0.98 for Case I; *w_AABB_* = *w_aabb_* = 1, *w_AaBB_* = *w_aaBb_* = 0.99, *w_AABb_* = *w_Aabb_* = 0.5, *w_aaBB_* = 0.98, *w_AAbb_* = 0.5, and *w_AaBb_* = 0.5 for Case II; *w_AABB_* = *w_aabb_* = 1, *w_AaBB_* = *w_aaBb_* = 0.99, *w_AABb_* = *w_Aabb_* = 0.1, *w_aaBB_* = 0.98, *w_AAbb_* = 0.1, and *w_AaBb_* = 0.1for Case III. Other parameter settings are the recombination rate = 5%, the immigration rate of pollen *m_P_* = 0.08 and seeds *m_S_* = 0.04, and the effective population size = 50. The genotypic frequencies in the continent and initial island populations are 0.1225 for *AABB*, 0.105 for *AABb*, 0.0225 for *AAbb*, 0.105 for *AaBB*, 0.245 for *AB/ab*, 0.045 for *ab/aB*, 0.105 for *Aabb*, 0.0225 for *aaBB*, 0.105 for *aaBb*, and 0.1225 for *aabb*. The steady-state results (mean ± *S_d_*) are obtained from 10000 independent simulation runs.

The above examples indicate that zygotic and gametic LDs have different responding patterns to natural selection. The cumulative selection can enhance zygotic LDs and other covariances in the additive-viability selection model. One striking result is that epistatic selection at the diploid level can produce zygotic LDs that are greater than or comparable to gametic LD. This pattern can be used to detect the epistatic selection process in natural populations.

### Analytical Theory

To further understand the evolution of zygotic LDs, I derive the analytical theory in a linear-additive-viability model with weak selection and random mating (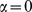). The gametic fitness in pollen and ovules is decomposed as 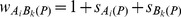 and 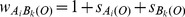 where 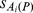 and 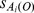 are the selection coefficients for allele 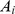 in pollen and ovules, respectively; 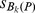 and 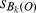 are the selection coefficients for allele 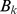 in pollen and ovules, respectively. The genotypic fitness in the sporophyte stage is expressed as 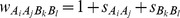 where 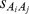
and 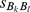
are the selection coefficients for genotypes 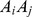 and 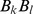, respectively.

With the weak selection, all items containing the second or higher orders of selection coefficients are neglected. The immigration rates of seeds and pollen are assumed to be small. The items containing the second or higher orders of the migration rate (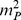, 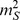, or 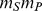, or higher orders) or the products of the migration rate with selection coefficients (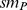 or 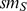) are neglected. Again, notation for the alleles and subscripts in the exact model is changed as *A* for 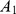, *a* for 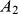, *B* for 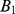, and *b* for 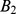. Selection coefficients are set as 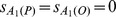, 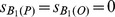, 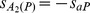, 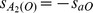, 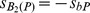, and 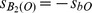. Alleles *a* and *b* are maladaptive in the island population. Let 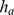 and 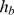 are the degrees of dominance at loci *A* and *B*, respectively. Selection coefficients for genotypes are set as 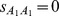, 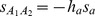, and 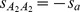 for locus *A*; and 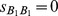, 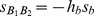, and 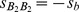 for locus *B.*

From Eqs. (A1) ∼ (A5) in Appendix S1, the deterministic changes in allelic frequency (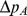 and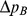), gametic (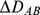) and four independent zygotic LDs (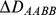, 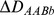, 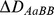, and 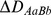), can be derived. Other functions of zygotic LDs can be calculated once the four independent zygotic LDs are available. After genetic drift, the means for the per-generation changes in allelic frequency, gametic and zygotic LDs, can be derived using the conventional approach [49] (Appendix S2). Note that one additional factor 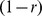
times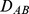 in the formulae in Appendix S2 is because 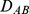 is termed from the preceding adults in a plant life cycle (one generation difference between adults and pollen and ovules; [41]).

Let 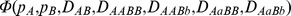
be the steady-state pdf at the two linked loci so that 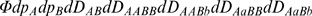 represents the expected number of two loci having the allele frequencies, gametic and zygotic LDs within the intervals (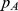,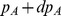), (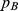,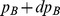), …, and (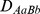, 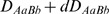 ), respectively. Expectation of each individual variable can be calculated in theory from pdf 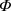. For instance, an expectation of gametic LD can be obtained by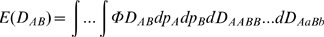. For a stationary distribution of a function of seven variables 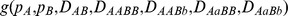, the Kolmogorov backward equation can be derived in the following expression [12], [50]:
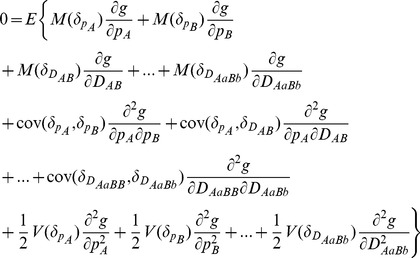
(3)


Notation *E* in Eq. (3) means expectation with respect to pdf 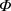, the same meaning as in the preceding section except that its calculation is based on numerical simulations.

In Eq. (3), there are seven items with the average change coefficients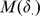, seven items with the variance coefficients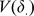, and twenty-one items with the covariance coefficients. Appendix S3 gives the expressions for the variances of per-generation changes in allelic frequency, gametic and zygotic LDs, and all possible covariances among these per-generation changes.

With the diffusion model, the expectations of zygotic LDs and the covariances between gametic and zygotic LDs or between different zygotic LDs can be calculated in theory. However, the algebraic deduction remains complicated when the joint effects of selection, migration, and genetic drift are considered. Here, I consider two specific cases. One case is that locus *A* is selective while locus *B* is neutral, with an emphasis on genetic hitchhiking effects [51], [52]. The other case is that both loci are neutral, with an emphasis on the effects of linkage distance.

**Genetic Hitchhiking.** How genetic hitchhiking effects evolve zygotic LDs is an important issue for studying the pattern of genotypic diversity along chromosomes. This may provide a genetic basis for forming zygotic LD blocks, analogous to the gametic LD blocks along chromosomes. Suppose that locus *A* is mainly subject to the balance between the effects of selection and immigration. The genetic drift effects are negligible for locus *A*. Locus *B* is subject to the balance among the effects of immigration, genetic drift, and recombination with locus *A*. This consideration is similar to the previous studies in examining associative overdominance or genetic hitchhiking effects on spreading neutral nuclear/organelle genes [44], [53], [54], [55]. All items with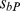, 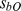, and 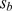 are eliminated for the average per-generation changes in allelic frequency, gametic and zygotic LDs in the formulae in Appendix S2. The variances for the per-generation changes in allelic frequency 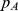 and all covariances between 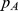 and gametic LD or between 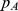 and zygotic LDs are removed in the formulae in Appendix S3, but the remaining expressions hold except that the steady-state 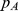 is known. Similarly, the items containing 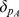,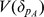, and the covariances between 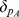 and gametic LD or between 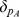 and different zygotic LDs in Eq. (3) are removed.

The steady-state equation for allelic frequency at locus *A* can be obtained by setting 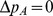, the same as setting 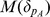 = 0, and 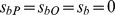 in Appendix S2, i.e. 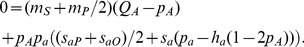
(4)


The steady-state allelic frequency can be numerically calculated from the above cubic equation, given the condition of 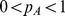 and other parameters. Like Ohta and Kimura [44], denote 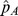
or 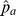 as the known frequencies calculated from Eq. (4). It can be seen that selection in the gametophyte and sporophyte stages is compounded in the case of *h_a_* = 1/2.

To calculate the expectations of the steady-state zygotic LDs and other types of covariances from Appendices S2 and S3, the following fourteen expectations are required: 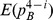
(*i* = 0, 1, 2, 3), 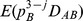
(*j* = 0,1,2,3), 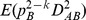
(*k* = 0,1,2), 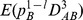
(*l* = 0,1), and 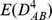. Expectations of a few low-order functions can be analytically derived. For instance, letting 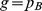 and 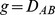 separately in Eq. (3), I can obtain:



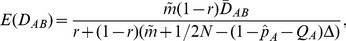
(5)

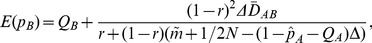
(6)where 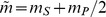, the joint migration rate, and 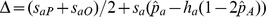, the selection component at locus *A*. Eq. (6) indicates the dependence of the allelic frequency at locus *B* on the allelic frequency at locus *A*.

Substitution of *g* in Eq. (3) by three functions, 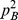, 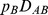, and 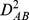, can yield three equations for calculating 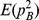, 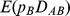, and 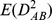:



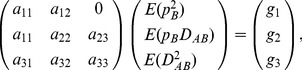
(7)


where 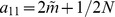, 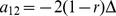,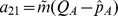, …, and 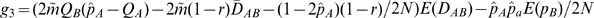.

Expectations of the remaining nine functions can be numerically calculated using *Mathematica* tool [56] by substituting *g* in Eq. (3) with 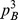, 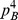, 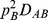, 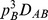, 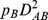, 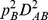, 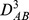, 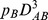, and 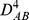, respectively. These calculations are not shown here.

With the availability of the above fourteen expectations, the expectations of some lower or the same order functions can be indirectly calculated. For instance, I can obtain 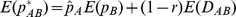 and 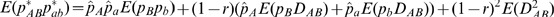. The expectation of any steady-state zygotic LD, 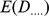, can be calculated by substituting *g* in Eq. (3) with *D_…._* (one of the four independent zygotic LDs), resulting in 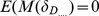. For instance, 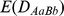
can be calculated by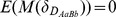 from Appendix S2, i.e.
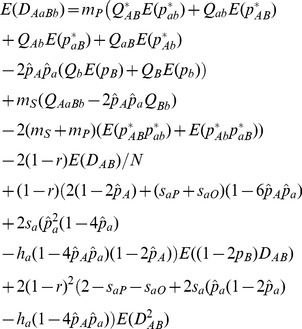
(8)


Eqs. (5) and (8) indicate that effects of seed and pollen flow are compounded in generating gametic LD, but can be separated in generating zygotic LDs.

The expectations of the steady-state variances of any zygotic LDs can be calculated using Fisher’s delta method by omitting all items containing 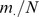, 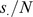, and higher orders. It is shown that these expectations can be calculated from the expectations of the variances of the per-generation change in zygotic LD in Appendix S3, i.e. 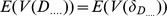
with a sufficient accuracy (Appendix S4; [12]). For instance, 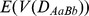 can be calculated from 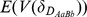, i.e.
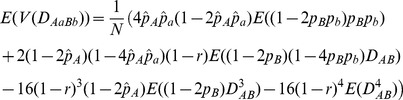
(9)


The expectation of any steady-state covariance between gametic and different zygotic LDs can be calculated using Fisher’s delta method by omitting all items with 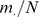, 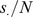, and higher orders. It is also shown that this expectation can be calculated from the expectation of the covariance in its per-generation change in Appendix S3, i.e. 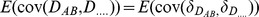 with a sufficient accuracy (Appendix S4). For instance, 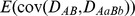 can be calculated from 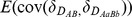
in Appendix S3, i.e.
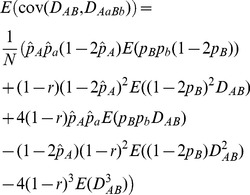
(10)


Similarly, expectations of other covariances in Appendix S3, such as the covariances between different zygotic LDs, can be calculated in the way similar to the above deductions. Expectations of high-order LDs, such as 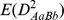, can be numerically calculated using multiple equations derived by substituting *g* in Eq. (3) with 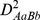 and other high-order LDs. This needs more extensive algebraic analyses, and is not explored further.

Simulations confirm that the above analytical model performs well. For instance, consider the same parameter settings as in Figure 1 for the genotypic frequencies in the continent and island populations, 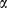  = 0, 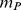 = 0.08 and 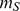 = 0.04, *N* = 100, 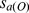 = 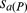 = 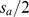 = 0.04, and *h_a_*  = 0.5. Gametic and zygotic LDs and other covariances can reach steady-state distributions (∼50^th^ generation; data not shown here), reflecting the equilibrium among the effects of migration, genetic drift, and genetic hitchhiking. All analytical predictions are distributed within the ranges of one-standard deviations of the simulation results (Table 3).

**Table 3:** Comparison between the simulation results and analytical model predictions under genetic hitchhiking effects*

	MC simulations	Analytical model
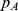	0.7165±0.0716	0.7071
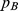	0.5916±0.0915	0.5509
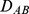	0.0777±0.0355	0.0536
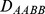	0.0732±0.0405	0.0465
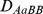	–0.0533±0.0347	–0.0317
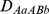	–0.0367±0.0317	–0.0169
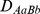	0.0419±0.0375	0.0202
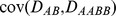	2.011×10^−4^±4.323×10^−5^	2.018×10^−4^
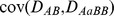	–1.463×10^−4^±3.994×10^−5^	–1.389×10^−4^
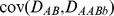	–1.059×10^−4^± 5.793×10^−5^	–7.188×10^−5^
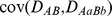	1.541×10^−4^±6.185×10^−5^	1.032×10^−4^
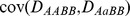	–3.918×10^−4^±7.381×10^−5^	–4.125×10^−4^
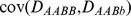	–3.496×10^−4^±8.167×10^−5^	–3.514×10^−4^
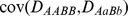	3.140×10^−4^±7.641×10^−5^	3.123×10^−4^
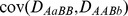	3.278×10^−4^±7.803×10^−5^	3.217×10^−4^
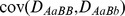	–3.310×10^−4^±9.101×10^−5^	–3.366×10^−4^
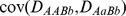	–4.654×10^−4^±5.826×10^−5^	–5.191×10^−4^

* Parameter settings are the immigration rate of pollen *m_P_* = 0.08 and seeds *m_S_* = 0.04, the effective population size = 100, the selection coefficients in the gametophyte and sporophyte stages *s_aO_* = *s_aP_* = *s_a_*/2 = 0.04, and the degree of dominance  = 0.5. The genotypic frequencies in the continent and the initial island populations are 0.1225 for *AABB*, 0.105 for *AABb*, 0.0225 for *AAbb*, 0.105 for *AaBB*, 0.245 for *AB/ab*, 0.045 for *ab/aB*, 0.105 for *Aabb*, 0.0225 for *aaBB*, 0.105 for *aaBb*, and 0.1225 for *aabb*. The steady-state simulation results (mean ± *S_d_*) are obtained from 10000 independent runs.

**Figure 5:**
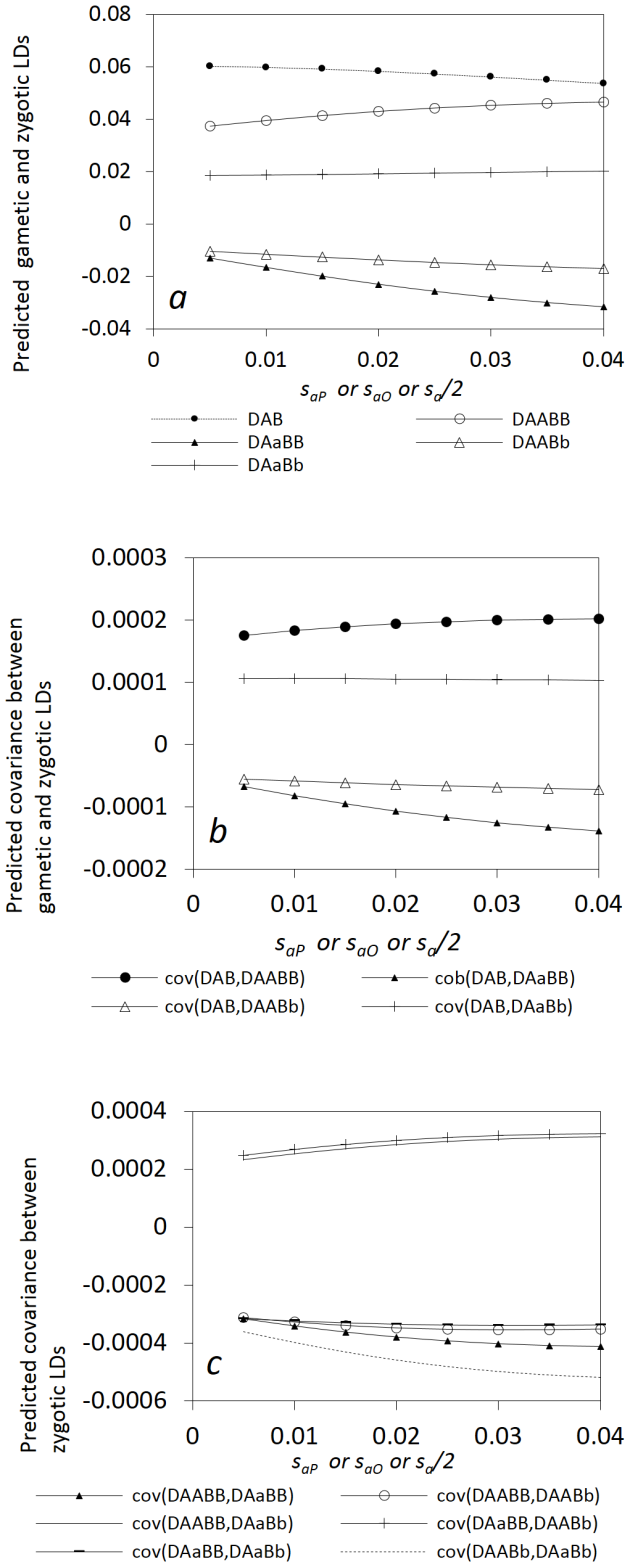
Genetic hitchhiking effects on the steady-state gametic and zygotic LDs, and other types of covariances. Gametic and zygotic LDs (a); covariances between gametic and zygotic LDs (b); and covariances between distinct zygotic LDs (c). Results are obtained from the analytical model in the section of Analytical Theory. Parameter settings are the immigration rate of pollen *m_P_* = 0.08 and seeds *m_S_* = 0.04, the effective population size = 100, the selection coefficients in the gametophyte and sporophyte stages *s_aO_* = s_a*P*_ = *s_a_*/2, and the degree of dominance  = 0.5. The genotypic frequencies in the continent and initial island populations are 0.1225 for *AABB*, 0.105 for *AABb*, 0.0225 for *AAbb*, 0.105 for *AaBB*, 0.245 for *AB/ab*, 0.045 for *ab/aB*, 0.105 for *Aabb*, 0.0225 for *aaBB*, 0.105 for *aaBb*, and 0.1225 for *aabb*.

Figure 5 shows that genetic hitchhiking effects can produce different patterns among gametic and zygotic LDs and other covariances. The expected neutral allelic frequency, *E*(*p_B_*), increases as the frequency of favorite allele *A* increases with the selection coefficient. 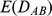 gradually decreases while *E*(*D_AABB_*) gradually increases with the selection coefficient (Figure 5a). *E*(*D_AaBb_*) slightly increases while both *E*(*D_AaBB_*) and *E*(*D_AABb_*) decrease with the selection coefficient. The covariances between gametic and zygotic LDs for the genotypes with heterozygotes at one locus or two loci gradually decrease with the selection coefficient, except *E*( *cov*(*D_AB_*, *D_AABB_*)) showing a different pattern (Figure 5b). The covariances between the zygotic LDs for the genotypes with one common genotype at a locus decrease with the selection coefficient, while the covariances between the zygotic LDs for the genotypes without a common genotype at one locus (*E*(*cov*(*D_AABB_*, *D_AaBb_*)) and *E*(*cov*(*D_AaBB_*, *D_AABb_*))) increase with the selection coefficient (Figure 5c). The covariances between the zygotic LDs for the genotypes with a common genotype at the selective locus, i.e. *E*(c*ov*(*D_AABB_*, *D_AABb_*)) and *E*(*cov*(*D_AaBB_*, *D_AaBb_*)), are less sensitive to selection than the covariances between the zygotic LDs for the genotypes with a common genotype at the neutral locus, i.e. *E*(*cov*(*D_AABB_*, *D_AaBB_*)) and *E*(*cov*(*D_AABb_*, *D_AaBb_*)).

The above results indicate that the gametic LD can have a similarly changing pattern to some zygotic LDs with the selection pressure. This provides the genetic basis of using zygotic LDs to describe genetic hitchhiking effects at the diploid level. Furthermore, the covariances between gametic and zygotic LDs or between distinct zygotic LDs are informative to indicate genetic hitchhiking effects.

**Neutral Process.** How zygotic LDs evolve in a purely neutral process forms another important issue to study the pattern of genotypic diversity along chromosomes since most molecular population evolution is governed by the neutral process. This also provides theoretical perception of using zygotic LD to reveal the structure of genomic diversity. Suppose that both loci are subject to the balance among the effects of genetic drift, recombination, and immigration. All items with selection coefficients are removed in the formulae in Appendix S2, but all the formulae in Appendix S3 remain unaltered. To assess the steady-state zygotic LDs and other covariances (Appendices S2 and S3), I need to calculate the following fifty-four expectations: 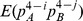
(*i, j* = 0, 1, 2, 3,4; except *i* = *j* = 4), 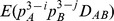
(*i, j* = 0,1,2,3), 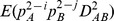
(*i, j* = 0,1,2), 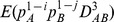
(*i, j* = 0,1), and 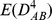. These expectations can be numerically calculated with *Mathematica* tool in different equations or different groups of equations.

Letting 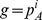
(*i* = 1, 2, 3, 4) in Eq. (3), I can obtain



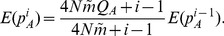
(11)


The *i*th moment of allelic frequency is the same as that derived under a neutral process for individual loci since LD does not affect allelic frequency distribution. Let 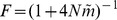
(an inbreeding coefficient in the island population) [57], which is equal to 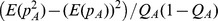, analogous to the population differentiation coefficient *F_st_* in the classical island model for plants [58]. Eq. (11) can be rewritten as 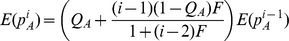, the same as Wright’s expression except for plant species here ([11], p.450). Eq. (11) represents the steady-state moments of allelic frequency under the balance of migration-genetic drift, different from Robertson’s results in a progressive inbreeding process [59]. Replacement of subscripts *A* with *B* in Eq. (11) yields 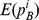 (*i*  = 1, 2, 3, 4).

Substituting 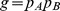 and 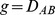 separately in Eq. (3) to yield two equations, I can obtain



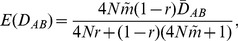
(12)

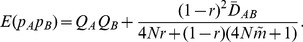
(13)


Eq. (12) indicates that the expectation of gametic LD is equal to zero in the absence of LD in migrants, such as in a completely isolated population. Eq. (13) indicates that the expectation of joint allele frequencies at two loci is related to the gametic LD in migrants (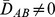) although expectations of individual allele frequencies are independent from each other (Eq. (11)).

Expectations of the remaining forty-four functions can be calculated in the following steps. Substitution of *g* in Eq. (3) by functions 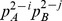
(*i*, *j* = 0,1; except *i* = *j* = 1), 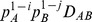 (*i*, *j* = 0,1; except *i* = *j* = 1), and 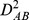 can yield seven equations that can be used to numerically calculate their expectations: 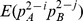
(*i*, *j* = 0,1; except *i* = *j* = 1), 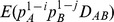
(*i*, *j* = 0,1; except *i* = *j* = 1), and 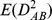. Substitution of *g* in Eq. (3) by functions 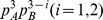, 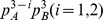, 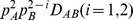, 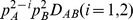, 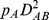, and 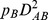, yields ten equations to calculate their expectations. Substitution of *g* in Eq. (3) with two functions 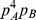 and 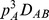 yields two equations to calculate 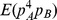 and 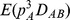. Substitution of *g* in Eq. (3) with two functions 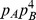, and 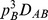 yields two equations to calculate 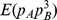 and 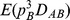. Substitution of *g* in Eq. (3) with functions 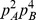, 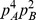, 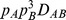, 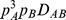, 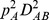, and 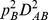, yields six equations to calculate their expectations: 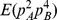, …, and 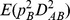. Finally, substitution of *g* in Eq. (3) with the remaining seventeen functions 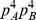, …, and 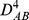, yields seventeen equations to calculate their expectations: 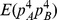, …, and 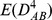.

The above order of *g* substitutions with different functions is sequentially arranged since calculations of the expected functions in the later equations need the expectations of the functions derived from the former equations. All these calculations can be done using *Mathematica* equation solution tool. Expectations of high-order LD functions, such as 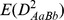, can also be calculated with additional equations by setting *g* in Eq. (3) with different functions. These are not explored further.

Once the expectations of the above fifty-four functions are available, the expectations of lower or the same order functions can be indirectly calculated. For instance, I can obtain 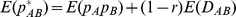 and 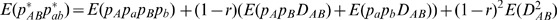. The expectations of all possible zygotic LDs, the variances of zygotic LDs, and the covariances among different LDs at the steady state can be calculated according to Appendices S2 and S3.

For instance, the expectation of steady-state zygotic LDs for the genotype with double heterozygotes (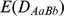 from 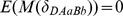), its variance (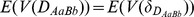), and its covariance with gametic LD (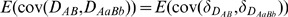) are given by
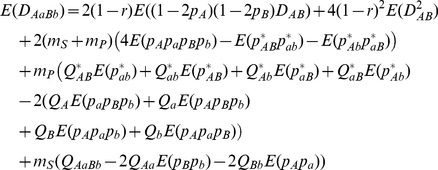
(14)

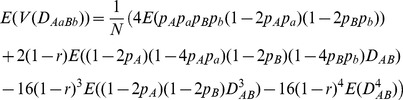
(15)

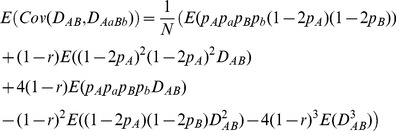
(16)


Simulations confirm that the above analytical model performs well. The gametic and zygotic LDs and other covariances between two neutral loci can quickly reach steady-state distributions, reflecting the equilibrium among the effects of migration, recombination, and genetic drift. All analytical results are distributed within the range of one standard deviation of the simulation results (Table 4). Simulations also confirm that the expectations of *D_AABb_* and *D_AaBB_* and their covariances with gametic or other zygotic LDs are the same because both loci are neutral. This symmetry may help to reduce the number of expectations of distinct functions in theory.

**Table 4:** Comparison between the simulation results and analytical model predictions under a neutral process*

	MC simulations	Analytical model
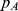	0.5079±0.0898	0.5
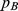	0.5102±0.0913	0.5
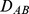	0.0974±0.0399	0.0615
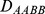	0.0623±0.0363	0.0360
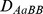	–0.0251±0.0312	–0.0100
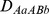	–0.0256±0.0312	–0.0100
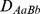	0.0462±0.0383	0.0200
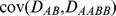	1.671×10^−4^±3.842×10^−5^	1.689×10^−4^
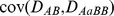	–0.909×10^−4^±4.71×10^−5^	–0.634×10^−4^
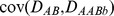	–0.916×10^−4^±4.67×10^−5^	–0.634×10^−4^
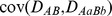	1.774×10^−4^±5.876×10^−5^	1.269×10^−4^
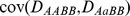	–3.164×10^−4^±8.517×10^−5^	–2.921×10^−4^
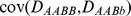	–3.167×10^−4^±8.527×10^−5^	–2.921×10^−4^
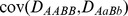	2.318×10^−4^±8.527×10^−5^	2.193×10^−4^
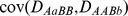	2.575×10^−4^±8.78×10^−5^	2.267×10^−4^
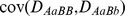	–2.977×10^−4^±1.002 ×10^−4^	–3.237×10^−4^
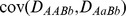	–2.949×10^−4^±0.990×10^−4^	–3.237×10^−4^

* Parameter settings are the immigration rate of pollen *m_P_* = 0.08 and seeds *m_S_* = 0.04, the effective population size = 100, and the recombination rate  = 0.05. The genotypic frequencies in the continent and the initial island populations are 0.1225 for *AABB*, 0.105 for *AABb*, 0.0225 for *AAbb*, 0.105 for *AaBB*, 0.245 for *AB/ab*, 0.045 for *ab/aB*, 0.105 for *Aabb*, 0.0225 for *aaBB*, 0.105 for *aaBb*, and 0.1225 for *aabb*. The steady-state simulation results (mean ± *S_d_*) are obtained from 10000 independent runs.

**Figure 6:**
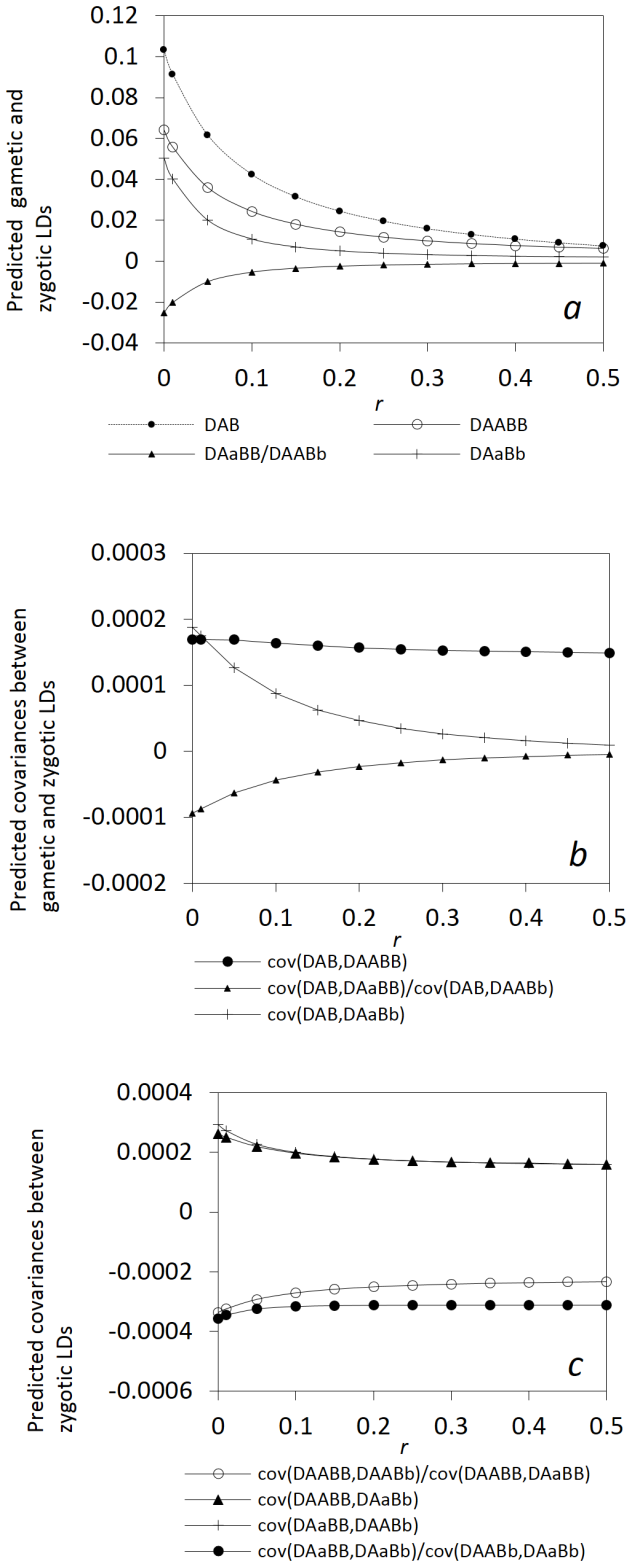
Effects of the linkage distance on the steady-state gametic and zygotic LDs and other types of covariances in a neutral process. Gametic and zygotic LDs (a); covariances between gametic and zygotic LDs (b); and covariances between distinct zygotic LDs (c). Results are obtained from the analytical model in the section of Analytical Theory. Parameter settings are the immigration rate of pollen *m_P_* = 0.08 and seeds *m_S_* = 0.04, the effective population size = 100, the selection coefficients in the gametophyte and sporophyte stages *s_aO_* = *s_aP_* = *s_a_* = 0, and the degree of dominance  = 0.0. The genotypic frequencies in the continent and initial island populations are 0.1225 for *AABB*, 0.105 for *AABb*, 0.0225 for *AAbb*, 0.105 for *AaBB*, 0.245 for *AB/ab*, 0.045 for *ab/aB*, 0.105 for *Aabb*, 0.0225 for *aaBB*, 0.105 for *aaBb*, and 0.1225 for *aabb*.

Figure 6 shows that different patterns exist for the expectations of gametic and zygotic LDs and other covariances with the recombination rate. 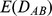 decreases faster than the absolute expectations of zygotic LDs with the recombination rate in addition to their inequality in magnitude (Figure 6a). Figure 6b shows that the absolute expectations of the covarainces between gametic and zygotic LDs gradually decrease with the recombination rate for the genotypes with heterozygotes at one locus or two loci. *E*(*cov*(*D_AB_*, *D_AABB_*)) slightly decreases with the recombination rate, but does not approach zero in the presence of immigration that maintains gametic and zygotic LDs. Figure 6c shows that the covariances between different zygotic LDs are generally not as sensitive as some covariances between gametic and zygotic LDs to the change of linkage distance. *E*(*cov*(*D_AABB_*, *D_AaBb_*)) and *E*(*cov*(*D_AaBB_*, *D_AABb_*)) slightly decrease with the recombination rate, while the covariances between the zygotic LDs of the genotypes with one common genotype at a locus slightly increase with the recombination rate.

The above results indicate that a neutral process can generate a similar pattern between zygotic and gametic LDs along chromosomes, with strong LDs within short distances and weak LDs within long distances. The covariances between gametic and zygotic LDs or between distinct zygotic LDs are relatively insensitive to the linkage distance.

## Discussion

In this study, I have developed the evolutionary theory of zygotic LDs in a local plant population, complementing the previous theories that mainly focus on the statistical issues [5], [6], [9]. The theory shows that evolutionary forces can generate different patterns among gametic and zygotic LDs, the covariances between gametic and zygotic LDs, and the covariances between different zygotic LDs. Zygotic LDs can be greater or smaller than, or comparable to gametic LD, depending on the major ecological and evolutionary processes involved in a local population. The covariances between gametic and zygotic LDs are more sensitive to the effects of mating system, linkage distance, and genetic drift, than to the effects of seed and pollen flow and selection. The covariances between different zygotic LDs are relatively robust to the change in gene flow, selection, and genetic distance, but sensitive to the genetic drift effects and mating system. Consistent patterns exist for the covariances between zygotic LDs for the genotypes with a common genotype at one locus, or for the genotypes without any common genotype at each locus. These similarities and differences suggest the potential utility of zygotic LDs in revealing the ecological and evolutionary processes underlying the pattern of population genomic diversity at the diploid level.

**Figure 7:**
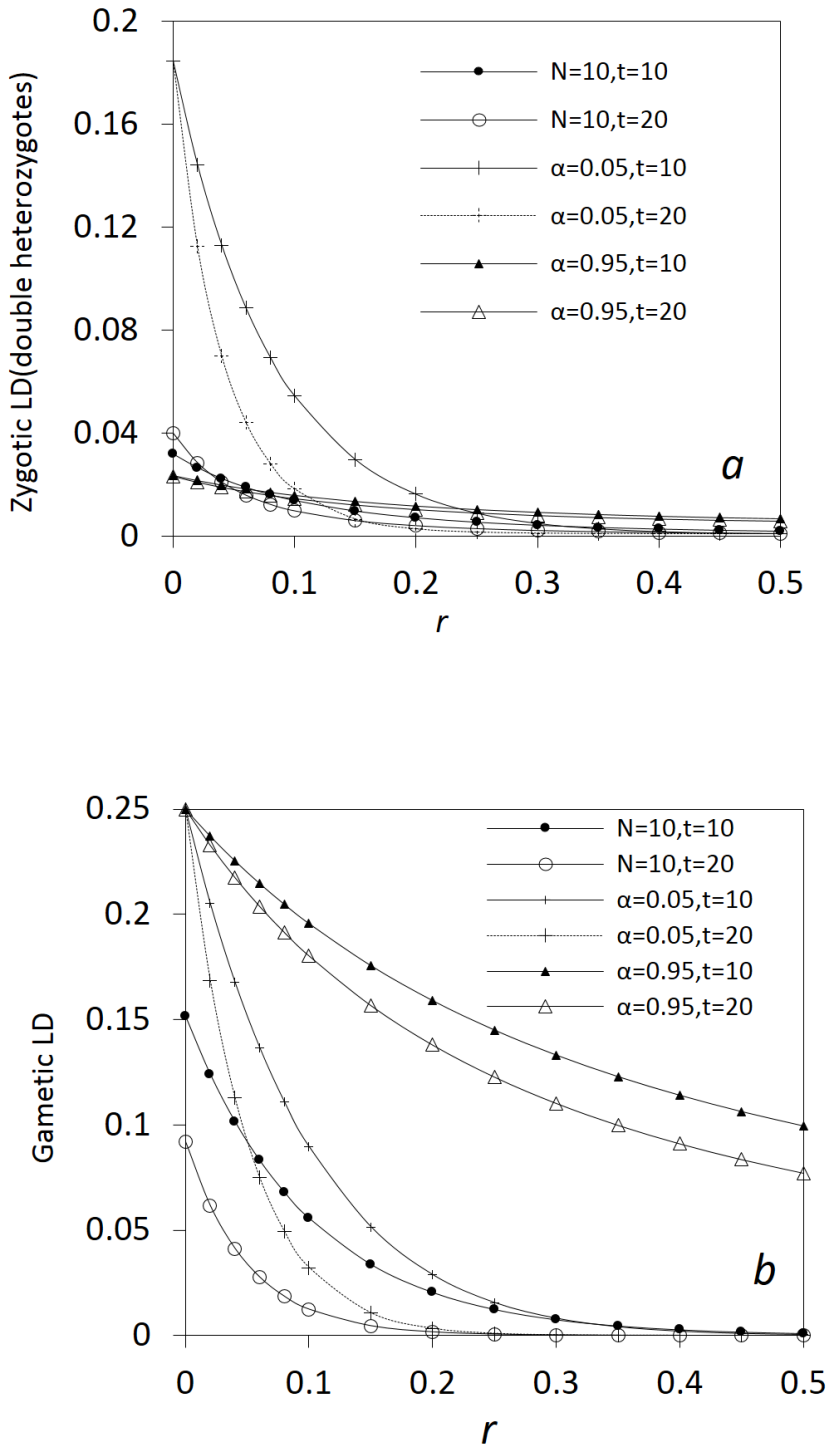
A comparison of transient gametic and zygotic LDs in a finite isolated population versus in an infinite population. Zygotic LDs in the finite population are calculated by synthesizing the theories of Robertson [59] and Ohta and Kimura [12]: *N* = 10, *t* = 10, and *N* = 10, *t* = 20 in (a). Gametic LD in the finite population is calculated from Hill and Robertson [8]: *N* = 10, *t* = 10, and *N* = 10, *t* = 20 in (b). Gametic and zygotic LDs in the infinite population are calculated from Weir and Cockerham [4]: *α* = 0.05, *t* = 10; *α* = 0.05, *t* = 20; *α* = 0.95, *t* = 10; and *α* = 0.95, *t* = 20 in (a) and (b). The initial settings for the finite population are *N* = 10 and the frequency of double heterozygotes (coupling)  = 1 (gametic LD = 0.25 and the allelic frequency at each diallelic locus = 0.5). The initial setting for the infinite population is the frequency of double heterozygotes (coupling)  = 1 (gametic LD = 0.25 and the allelic frequency at each diallelic locus = 0.5).

It is important to understand that in a pure drift process, LD is transient in a completely isolated population of random mating. Expectations of both gametic and zygotic LDs are zero although the expectations of their squared values are nonzero [8], [44], [60]. Zygotic LDs are smaller than gametic LDs in magnitude, but decay more slowly than gametic LDs with time [12], [46]. This is because the genotypic association may primarily arise from the effects other than the linkage distance although the linkage distance can affect the frequencies of genotypic recombinants. Weir and Cockerham[4] and Cockerham and Weir [3] have decomposed gametic and zygotic LDs in terms of descent measures for an infinite population with a mixed mating system. Both gametic and zygotic LDs decay faster in an infinite than in a finite population within a short linkage distance when the genetic drift effects are in the same order as the selfing rate (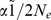; e.g., Figure 7). A predominantly selfing population reduces the rates of decay of both gametic and zygotic LDs. When additional driving forces are involved, the above “null” expectation and the rates of decay in gametic and zygotic LDs could be changed [1],[2].

Note that the theory only addresses the constant immigration of seeds and pollen. In reality, a frequent situation is the stochastic migration of seeds and pollen due to the influences of biotic and abiotic factors [61], [62]. This occurs particularly when the source populations or the pollen and seed pools are unstable. Under this situation, the gametic and genotypic frequencies fluctuate in migrating seeds and pollen, and so do the gametic and zygotic LDs in migrants. Zygotic and gametic LDs can exhibit more fluctuations under the joint effects of genetic drift and stochastic migration. This can weaken the relationships between zygotic and gametic LDs. Nevertheless, the explored qualitative relationships between zygotic LDs and migration remain valid. How the stochastic migration of seeds and pollen affects the relative gametic and zygotic LDs remains unclear, and this forms a topic for further study.

Also, note that a plant mating system in a natural population may exhibit a dynamic property [25]. Mating system can be naturally changed through different ways [63], such as the change of pollen pool and the shift from wind to animal pollinations [64], [65]. Since zygotic LDs and other covariances are sensitive to the change of mating system (Figure 1), an unstable mating system enhances the fluctuation of these covariances. Nevertheless, the non-linear relationships between the selfing rate and zygotic LDs remain valid.

Apart from the above assumptions, the theory suggests several useful implications [1], [2]. First, the newly explored relationships between gametic and zygotic LDs under different evolutionary forces, not the purely statistical relationships [3], [5], [6], suggest their different or similar practical applications. Different patterns between gametic and zygotic LDs indicate that they can be applied for different purposes. Zygotic LDs provide additional information for inferring population history. Previous studies emphasize the use of gametic LD for this purpose [61], [66]. The present theory shows that zygotic LDs exhibit more diverse patterns in response to different driving forces, which can reinforce our inference on the major ecological and evolutionary processes.

The occurrence of a weak gametic LD combined with strong zygotic LDs suggests epistatic interactions at the diploid level (e.g., postzygotic isolation due to the Dobzhansky-Muller incompatibility [27], [28], [47]). The occurrence of a strong gametic LD combined with weak zygotic LDs suggests the involvement of non epistatic processes at the diploid level, including migration, linear-additive selection, and genetic drift processes. When both strong gametic and zygotic LDs arise from the tight linkage, they can be applied for the same purposes. For example, in analyzing normalized gametic and zygotic LDs in a human population, the same SNP markers exist when both gametic and zygotic LDs are very strong (say, the squares of normalized gametic and zygotic LDs >0.9; [67]). The relatively stable patterns in zygotic and gametic LDs and in covariances between gametic and zygotic LDs across multiple populations suggest the impacts of seed and pollen flow or weak selection. Patterns from multiple samples of a given population or from multiple different natural populations can strengthen such inferences.

Second, the theory provides a genetic basis of using zygotic LDs for QTL mapping that has been recently addressed [23]. A similar pattern between zygotic and gametic LDs with the linkage distance implies the common utility for QTL mapping. Zygotic LD-based QTL mapping can be conducted in nonrandom mating populations [23]. One caution is that spurious and unstable non-random associations can occur in natural populations under the influences of the driving forces other than the recombination process. This can influence the accuracy and precision of QTL mapping. QTL mapping based on the linkage maps from a single family, such as a half-sib family from a single tree or a full-sib family from a single cross, is not affected. However, the population-based linkage maps could be affected although this approach is commonly suggested to search for LDs within a short linkage distance at a finer scale [68], [69]. Thus, the patterns of zygotic LDs can be used to preliminarily screen markers for QTL mapping through a high criterion [46], or to effectively remove spurious LDs through a deliberate experiment [70]. This may improve QTL mapping with the population-based linkage maps.

Third, the theory aids in predicting the effects of seed and pollen flow on zygotic LDs in a local population. Previous studies use gametic LD to estimate gene flow in a specific case, such as in hybrid zones [71]. The present theory shows that gametic LD is more sensitive than zygotic LDs to either seed flow or pollen flow. Seed flow has greater effects than pollen flow on gametic LD. In natural populations of flowering plants, pollen flow is often more extensive than seed flow among mature populations, especially for the predominantly outcrossing species [72]. The cumulative effects on gametic LD from pollen flow could be substantial. The robust pattern of zygotic LDs to the impacts of seed or pollen flow enables their utility for inferring if gametic LD is generated by the forces other than migration. One extreme case is the admixture of two or more plant or animal populations, such as cross breeding, which results in the same consequence as that produced by a large proportion of immigrating seeds. This produces extensive gametic LDs rather than zygotic LDs [61], [66]. Only those tightly linked loci can maintain strong zygotic and gametic LDs [46]. Thus, the multilocus patterns of joint gametic and zygotic LDs can be used to judge if immigration is an important process to shape gametic LDs in local populations.

Fourth, the theory aids in assessing the selection mode (additive or epistatic) in the gametophyte and sporophyte stages in generating gametic and zygotic LDs. “Bulmer effects” mainly emphasize the impacts of selection on gametic LD [26], but gametic LD does not provide the information on the genotypic interaction at the diploid level. Extensive reports are recorded in the literature about the use of gametic LD for detecting selection signature along chromosomes [73]. So far, zygotic LDs have not been applied to detecting the genetic basis of adaption at the diploid level. In the linear additive-viability model, selection from the two stages is compounded. Gametic LD is greater than zygotic LDs in magnitude because selection affects gametic LD at each stage but affects zygotic LDs only in the sporophyte stage, similar to the effects of haploid pollen and diploid seed flow. However, in the presence of epistatic selection at the diploid level, some genotypes have zygotic LDs larger than gametic LD while other genotypes have zygotic LDs smaller than gametic LD. Such divergent patterns can aid in our inference on epistatic selection. One typical situation is a natural hybrid zone (a tension zone)[29] where epistatic selection can cause zygotic LDs greater than gametic LD [41], which provides the information complementary to two non-allele interaction at the haploid level [27],[28], [30]. The joint patterns of gametic and zygotic LDs can be used to infer the selection mode (additive or epistatic) at the diploid level.

In addition, the genotypic interaction on fitness may arise from the dominance by dominance effects for *D_AaBb_*, or the additive by dominance effects for *D_AABb_* or *D_AaBB_*, or the additive by additive effects for *D_AABB_* at two loci. One further study is to assess the genetic mechanisms of these epistases in distinct zygotic LDs at the sporophyte stage.

Finally, it is of interest to discuss the utility of the covariances between distinct zygotic LDs since few studies have examined such high-order LDs [9], [10]. The present theory suggests one robust property of these high-order LDs, i.e. the presence of a consistent pattern for the genotypes with one common genotype at one locus or for the genotypes without any common genotype at each locus. This property can be used to effectively determine the impacts from migration, recombination, and additive weak selection, and to assess the effects of effective population size and/or a mating system. Given a stable effective population size and a stable mating system, a significant bias from the robust property implies epistatic selection (Table 2) or very diverse selection systems among genotypes. This requires further empirical verification with appropriate data collections.

## Supporting Information

Appendix S1 **Derivations of genotypic frequencies**
(DOC)

Appendix S2 **Average per-generation changes in allelic frequency, gametic and zygotic LDs**
(DOC)

Appendix S3 **Variances for the per-generation changes in allelic frequency, gametic and zygotic LDs**
(DOC)

Appendix S4 **Expectations of variances of zygotic LDs and the covariances between gametic and zygotic LDs**
(DOC)
