# Supplementary material for: Evolution of Zygotic Linkage Disequilibrium in a Finite Local Population
Source: PLoS One. 2013 Nov 27;8(11):e80538. doi: 10.1371/journal.pone.0080538 (PMC3842346; doi:10.1371/journal.pone.0080538)
Supplement: Appendix S1 — Derivations of genotypic frequencies (DOC) [file pone.0080538.s001.doc]

**Appendix S1 Derivations of genotypic frequencies**

Following the notation denoted in the main text, let$f_{A_{i}B_{k}(P1)}=w_{A_{i}B_{k}(P)}/(w_{A_{i}B_{1}(P)}+w_{A_{i}B_{2}(P)})$ and$f_{A_{i}B_{k}(P2)}=w_{A_{i}B_{k}(P)}/(w_{A_{1}B_{k}(P)}+w_{A_{2}B_{k}(P)})$ for pollen; and $f_{A_{i}B_{k}(O1)}=w_{A_{i}B_{k}(O)}/(w_{A_{i}B_{1}(O)}+w_{A_{i}B_{2}(O)})$ and$f_{A_{i}B_{k}(O2)}=w_{A_{i}B_{k}(O)}/(w_{A_{1}B_{k}(O)}+w_{A_{2}B_{k}(O)})$for ovules. These *f*’s variables are required to express gametic selection for the random-mating part. Let $c_{11}=c_{22}=(1-r)/2$, and $c_{12}=c_{21}=r/2$, the variables related to linkage phases. For the selfing part, let$f_{A_{i}B_{k}(P3)}=c_{ik}w_{A_{i}B_{k}(P)}/\sum_{i,k=1}^{2} c_{ik}w_{A_{i}B_{k}(P)}$and$f_{A_{i}B_{k}(P4)}=(1/2-c_{ik})w_{A_{i}B_{k}(P)}/\sum_{i,k=1}^{2} (1/2-c_{ik})w_{ik(P)}$ for pollen; and $f_{A_{i}B_{k}(O3)}=c_{ik}w_{A_{i}B_{k}(O)}/\sum_{i,k=1}^{2} c_{ik}w_{A_{i}B_{k}(O)}$and $f_{A_{i}B_{k}(O4)}=(1/2-c_{ik})w_{A_{i}B_{k}(O)}/\sum_{i,k=1}^{2} (1/2-c_{ik})w_{ik(O)}$for ovules.

According to the plant life cycle mentioned in the main text, the genotypic frequency after selection in the sporophyte stage, denoted by $p_{A_{i}A_{j}B_{k}B_{l}}^{**}$ ($=w_{A_{i}A_{j}B_{k}B_{l}}p_{_{A_{i}A_{j}B_{k}B_{l}}}^{*}/w$ ) for $A_{i}A_{i}B_{k}B_{l}$($i,j,k,l=1,2;i\leq j;k\leq l$), can be generally expressed in separate parts:

$$p_{A_{i}A_{i}B_{k}B_{k}}^{**}=(1-m_{S})\frac{w_{A_{i}A_{i}B_{k}B_{k}}}{w}\left((1-\alpha)\frac{w_{A_{i}B_{k}(P)}w_{A_{i}B_{k}(O)}}{w_{O}w_{P}}\left((1-m_{P})p_{A_{i}B_{k}}^{*\text{ }2}+m_{P}Q_{A_{i}B_{k}}^{*}p_{A_{i}B_{k}}^{*}\right)\right.$$

$$+\alpha\left(p_{A_{i}A_{i}B_{k}B_{k}}+f_{A_{i}B_{k}(O1)}f_{A_{i}B_{k}(P1)}p_{A_{i}A_{i}B_{1}B_{2}}+f_{A_{i}B_{k}(O2)}f_{A_{i}B_{k}(P2)}p_{A_{1}A_{2}B_{k}B_{k}}\right.$$

$$\left.\left.+f_{A_{i}B_{k}(O3)}f_{A_{i}B_{k}(P3)}p_{A_{1}B_{1}/A_{2}B_{2}}+f_{A_{i}B_{k}(O4)}f_{A_{i}B_{k}(P4)}p_{A_{1}B_{2}/A_{2}B_{1}}\right)\right)$$

+$m_{S}\frac{w_{A_{i}A_{i}B_{k}B_{k}}}{w}Q_{A_{i}A_{i}B_{k}B_{k}}$. (A1)

$$p_{A_{1}A_{2}B_{k}B_{k}}^{**}=(1-m_{S})\frac{w_{A_{1}A_{2}B_{k}B_{k}}}{w}\left((1-\alpha)\frac{w_{A_{1}B_{k}(P)}w_{A_{2}B_{k}(O)}}{w_{O}w_{P}}p_{A_{2}B_{k}}^{*}\left((1-m_{P})p_{A_{1}B_{k}}^{*}+m_{P}Q_{A_{1}B_{k}}^{*}\right)\right.$$

$$+(1-\alpha)\frac{w_{A_{2}B_{k}(P)}w_{A_{1}B_{k}(O)}}{w_{O}w_{P}}p_{A_{1}B_{k}}^{*}\left((1-m_{P})p_{A_{2}B_{k}}^{*}+m_{P}Q_{A_{2}B_{k}}^{*}\right)$$

$$+\alpha\left(\left(f_{A_{1}B_{k}(O2)}f_{A_{2}B_{k}(P2)}+f_{A_{1}B_{k}(P2)}f_{A_{2}B_{k}(O2)}\right)\right.p_{A_{1}A_{2}B_{k}B_{k}}$$

$$+\left(f_{A_{1}B_{k}(O3)}f_{A_{2}B_{k}(P3)}+f_{A_{1}B_{k}(P3)}f_{A_{2}B_{k}(O3)}\right)p_{A_{1}B_{1}/A_{2}B_{2}}$$

$$+\left(f_{A_{1}B_{k}(O4)}f_{A_{2}B_{k}(P4)}+f_{A_{1}B_{k}(P4)}f_{A_{2}B_{k}(O4)}\right)\left.p_{A_{1}B_{2}/A_{2}B_{1}}\right)$$

+$m_{S}\frac{w_{A_{1}A_{2}B_{k}B_{k}}}{w}Q_{A_{1}A_{2}B_{k}B_{k}}$. (A2)

$$p_{A_{i}A_{i}B_{1}B_{2}}^{**}=(1-m_{S})\frac{w_{A_{i}A_{i}B_{1}B_{2}}}{w}\left((1-\alpha)\frac{w_{A_{i}B_{1}(P)}w_{A_{i}B_{2}(O)}}{w_{O}w_{P}}p_{A_{i}B_{2}}^{*}\left((1-m_{P})p_{A_{i}B_{1}}^{*}+m_{P}Q_{A_{i}B_{1}}^{*}\right)\right.$$

$$+(1-\alpha)\frac{w_{A_{i}B_{2}(P)}w_{A_{i}B_{1}(O)}}{w_{O}w_{P}}p_{A_{i}B_{1}}^{*}\left((1-m_{P})p_{A_{i}B_{2}}^{*}+m_{P}Q_{A_{i}B_{2}}^{*}\right)$$

$$+\alpha\left(\left(f_{A_{i}B_{1}(O2)}f_{A_{i}B_{2}(P2)}+f_{A_{i}B_{1}(P2)}f_{A_{i}B_{2}(O2)}\right)\right.p_{A_{i}A_{i}B_{1}B_{2}}$$

$$+\left(f_{A_{i}B_{1}(O3)}f_{A_{i}B_{2}(P3)}+f_{A_{i}B_{1}(P3)}f_{A_{i}B_{2}(O3)}\right)p_{A_{1}B_{1}/A_{2}B_{2}}$$

$$+\left(f_{A_{i}B_{1}(O4)}f_{A_{i}B_{2}(P4)}+f_{A_{i}B_{1}(P4)}f_{A_{i}B_{2}(O4)}\right)\left.p_{A_{1}B_{2}/A_{2}B_{1}}\right)$$

+$m_{S}\frac{w_{A_{i}A_{i}B_{1}B_{2}}}{w}Q_{A_{i}A_{i}B_{1}B_{2}}$. (A3)

$$p_{A_{1}B_{1}/A_{2}B_{2}}^{**}=(1-m_{S})\frac{w_{A_{1}A_{2}B_{1}B_{2}}}{w}\left((1-\alpha)\frac{w_{A_{1}B_{1}(P)}w_{A_{2}B_{2}(O)}}{w_{O}w_{P}}\right.p_{A_{2}B_{2}}^{*}\left((1-m_{P})p_{A_{1}B_{1}}^{*}+m_{P}Q_{A_{1}B_{1}}^{*}\right)$$

$$+(1-\alpha)\frac{w_{A_{2}B_{2}(P)}w_{A_{1}B_{1}(O)}}{w_{O}w_{P}}p_{A_{1}B_{1}}^{*}\left((1-m_{P})p_{A_{2}B_{2}}^{*}+m_{P}Q_{A_{2}B_{2}}^{*}\right)$$

$$+\alpha\left(f_{A_{1}B_{1}(O3)}f_{A_{2}B_{2}(P3)}+f_{A_{1}B_{1}(P3)}f_{A_{2}B_{2}(O3)}\right)p_{A_{1}B_{1}/A_{2}B_{2}}$$

$$+\alpha\left(f_{A_{1}B_{2}(O4)}f_{A_{2}B_{1}(P4)}+f_{A_{1}B_{2}(P4)}f_{A_{2}B_{1}(O4)}\right)\left.p_{A_{1}B_{2}/A_{2}B_{1}}\right)$$

+$m_{S}\frac{w_{A_{1}A_{2}B_{1}B_{2}}}{w}Q_{A_{1}B_{1}/A_{2}B_{2}}$. (A4)

$$p_{A_{1}B_{2}/A_{2}B_{1}}^{**}=(1-m_{S})\frac{w_{A_{1}A_{2}B_{1}B_{2}}}{w}\left((1-\alpha)\frac{w_{A_{1}B_{2}(P)}w_{A_{2}B_{1}(O)}}{w_{O}w_{P}}\right.p_{A_{2}B_{1}}^{*}\left((1-m_{P})p_{A_{1}B_{2}}^{*}+m_{P}Q_{A_{1}B_{2}}^{*}\right)$$

$$+(1-\alpha)\frac{w_{A_{2}B_{1}(O)}w_{A_{1}B_{2}(O)}}{w_{O}w_{P}}p_{A_{1}B_{2}}^{*}\left((1-m_{P})p_{A_{2}B_{1}}^{*}+m_{P}Q_{A_{2}B_{1}}^{*}\right)$$

$$+\alpha\left(f_{A_{1}B_{1}(O3)}f_{A_{2}B_{2}(P3)}+f_{A_{1}B_{1}(P3)}f_{A_{2}B_{2}(O3)}\right)p_{A_{1}B_{1}/A_{2}B_{2}}$$

$$+\alpha\left(f_{A_{1}B_{2}(O4)}f_{A_{2}B_{1}(P4)}+f_{A_{1}B_{2}(P4)}f_{A_{2}B_{1}(O4)}\right)\left.p_{A_{1}B_{2}/A_{2}B_{1}}\right)$$

+$m_{S}\frac{w_{A_{1}A_{2}B_{1}B_{2}}}{w}Q_{A_{1}B_{2}/A_{2}B_{1}}$. (A5)
