# Supplementary material for: Evolution of Zygotic Linkage Disequilibrium in a Finite Local Population
Source: PLoS One. 2013 Nov 27;8(11):e80538. doi: 10.1371/journal.pone.0080538 (PMC3842346; doi:10.1371/journal.pone.0080538)
Supplement: Appendix S2 — Average per-generation changes in allelic frequency, gametic and zygotic LDs (DOC) [file pone.0080538.s002.doc]

**Appendix S2 Average per-generation changes in allelic frequency, gametic and zygotic LDs.**

According to Eqs. (A1) ~ (A5) in Appendix S1, the average per-generation changes are derived for allele frequency and gametic and zygotic LDs after genetic drift in a population of effective size *N*. All items with $m_{.}/N$, $s_{.}/N$, and higher-orders are neglected in deriving these formulae.

$$M(\delta_{p_{A}})=(m_{S}+m_{P}/2)(Q_{A}-p_{A})+p_{A}p_{a}\left((s_{aP}+s_{aO})/2+s_{a}(p_{a}-h_{a}(1-2p_{A}))\right)$$

$+(1-r)D_{AB}\left((s_{bP}+s_{bO})/2+s_{b}(p_{b}-h_{b}(1-2p_{B}))\right)$, (B1)

$$M(\delta_{p_{B}})=(m_{S}+m_{P}/2)(Q_{B}-p_{B})+p_{B}p_{b}\left((s_{bP}+s_{bO})/2+s_{b}(p_{b}-h_{b}(1-2p_{B}))\right)$$

$+(1-r)D_{AB}\left((s_{aP}+s_{aO})/2+s_{a}(p_{a}-h_{a}(1-2p_{A}))\right)$, (B2)

$+(1-r)D_{AB}\left((1-2p_{A})((s_{aP}+s_{aO})/2+s_{a}(p_{a}-h_{a}(1-2p_{A}))+(1-2p_{B})((s_{bP}+s_{bO})/2+s_{b}(p_{b}-h_{b}(1-2p_{B})))\right)$,

(B3)

$$M(\delta_{D_{AABB}})=(1-r)D_{AB}\left(2p_{A}p_{B}+(1-r)D_{AB}-1/N\right)-D_{AABB}+m_{P}\left(p_{AB}^{*}(Q_{AB}^{*}-p_{AB}^{*})+p_{A}^{2}p_{B}(p_{B}-Q_{B})+p_{A}p_{B}^{2}(p_{A}-Q_{A})\right)$$

$$+m_{S}\left(Q_{AABB}-p_{AB}^{*\text{ }2}+p_{A}^{2}(p_{B}^{2}-Q_{BB})+p_{B}^{2}(p_{A}^{2}-Q_{AA})\right)$$

$$-(s_{aP}+s_{aO})(1-r)D_{AB}\left(p_{A}p_{B}(1-3p_{a})-p_{a}(1-r)D_{AB}\right)$$

$$+s_{a}(1-r)D_{AB}\left(2p_{A}p_{a}p_{B}(1-2p_{A})+(1-2p_{A}p_{a})(1-r)D_{AB}+2h_{a}\left(p_{A}^{2}p_{B}(4p_{a}-1)+p_{A}(1-2p_{A})(1-r)D_{AB}\right)\right)$$

$$-(s_{bP}+s_{bO})(1-r)D_{AB}\left(p_{A}p_{B}(1-3p_{b})-p_{b}(1-r)D_{AB}\right)$$

$+s_{b}(1-r)D_{AB}\left(2p_{A}p_{B}p_{b}(1-2p_{B})+(1-2p_{B}p_{b})(1-r)D_{AB}+2h_{b}\left(p_{B}^{2}p_{A}(4p_{b}-1)+p_{B}(1-2p_{B})(1-r)D_{AB}\right)\right)$,

(B4)

$$M(\delta_{D_{AABb}})=2(1-r)D_{AB}\left(p_{A}(1-2p_{B})-(1-r)D_{AB}-1/N\right)-D_{AABb}$$

$$+m_{P}\left(p_{Ab}^{*}(Q_{AB}^{*}-p_{AB}^{*})+p_{AB}^{*}(Q_{Ab}^{*}-p_{Ab}^{*})+p_{A}^{2}(p_{B}(p_{b}-Q_{b})+p_{b}(p_{B}-Q_{B}))+2p_{B}p_{b}p_{A}(p_{A}-Q_{A})\right)$$

$$+m_{S}\left(Q_{AABb}-2p_{AB}^{*}p_{Ab}^{*}+p_{A}^{2}(2p_{B}p_{b}-Q_{Bb})+2p_{B}p_{b}(p_{A}^{2}-Q_{AA})\right)$$

$$-(s_{aP}+s_{aO})(1-r)D_{AB}\left(p_{A}(1-2p_{B})(1-3p_{a})+2p_{a}(1-r)D_{AB}\right)$$

$$+2s_{a}(1-r)D_{AB}\left(p_{A}p_{a}(1-2p_{B})(1-2p_{A})-(1-2p_{A}p_{a})(1-r)D_{AB}+h_{a}\left(p_{A}^{2}(1-2p_{B})(4p_{a}-1)-2p_{A}(1-2p_{A})(1-r)D_{AB}\right)\right)$$

$$-(s_{bP}+s_{bO})(1-r)D_{AB}\left(-p_{A}(1-6p_{B}p_{b})+(1-2p_{B})(1-r)D_{AB}\right)$$

$+2s_{b}(1-r)D_{AB}\left(p_{A}p_{b}^{2}(1-4p_{B})-p_{b}(1-2p_{B})(1-r)D_{AB}-h_{b}(1-4p_{B}p_{b})\left(p_{A}(1-2p_{B})-(1-r)D_{AB}\right)\right)$, (B5)

$$M(\delta_{D_{AaBB}})=2(1-r)D_{AB}\left(p_{B}(1-2p_{A})-(1-r)D_{AB}-1/N\right)-D_{AaBB}$$

$$+m_{P}\left(p_{aB}^{*}(Q_{AB}^{*}-p_{AB}^{*})+p_{AB}^{*}(Q_{aB}^{*}-p_{aB}^{*})+p_{B}^{2}(p_{A}(p_{a}-Q_{a})+p_{a}(p_{A}-Q_{A}))+2p_{A}p_{a}p_{B}(p_{B}-Q_{B})\right)$$

$$+m_{S}\left(Q_{AaBB}-2p_{AB}^{*}p_{aB}^{*}+p_{B}^{2}(2p_{A}p_{a}-Q_{Aa})+2p_{A}p_{a}(p_{B}^{2}-Q_{BB})\right)$$

$$-(s_{aP}+s_{aO})(1-r)D_{AB}\left(-p_{B}(1-6p_{A}p_{a})+(1-2p_{B})(1-r)D_{AB}\right)$$

$$+2s_{a}(1-r)D_{AB}\left(p_{B}p_{a}^{2}(1-4p_{A})-p_{a}(1-2p_{A})(1-r)D_{AB}-h_{a}(1-4p_{A}p_{a})\left(p_{B}(1-2p_{A})-(1-r)D_{AB}\right)\right)$$

$$-(s_{bP}+s_{bO})(1-r)D_{AB}\left(p_{B}(1-2p_{A})(1-3p_{b})+2p_{b}(1-r)D_{AB}\right)$$

$+2s_{b}(1-r)D_{AB}\left(p_{B}p_{b}(1-2p_{A})(1-2p_{B})-(1-2p_{B}p_{b})(1-r)D_{AB}+h_{b}\left(p_{B}^{2}(1-2p_{A})(4p_{b}-1)-2p_{B}(1-2p_{B})(1-r)D_{AB}\right)\right)$,

(B6)

and

$$M(\delta_{D_{AaBb}})=2(1-r)D_{AB}\left((1-2p_{B})(1-2p_{A})+2(1-r)D_{AB}-1/N\right)-D_{AaBb}$$

$$+m_{P}\left(p_{ab}^{*}(Q_{AB}^{*}-p_{AB}^{*})+p_{AB}^{*}(Q_{ab}^{*}-p_{ab}^{*})\right.+p_{aB}^{*}(Q_{Ab}^{*}-p_{Ab}^{*})+p_{Ab}^{*}(Q_{aB}^{*}-p_{aB}^{*})$$

$$+\left.2p_{B}p_{b}(p_{A}(p_{a}-Q_{a})+p_{a}(p_{A}-Q_{A}))+2p_{A}p_{a}(p_{B}(p_{b}-Q_{b})+p_{b}(p_{B}-Q_{B}))\right)$$

$$+m_{S}\left(Q_{AaBb}-2p_{Ab}^{*}p_{aB}^{*}-2p_{AB}^{*}p_{ab}^{*}+2p_{B}p_{b}(2p_{A}p_{a}-Q_{Aa})+2p_{A}p_{a}(2p_{B}p_{b}-Q_{Bb})\right)$$

$$-(s_{aP}+s_{aO})(1-r)D_{AB}\left(-(1-2p_{B})(1-6p_{A}p_{a})-2(1-2p_{A})(1-r)D_{AB}\right)$$

$$+2s_{a}(1-r)D_{AB}\left(p_{a}^{2}(1-2p_{B})(1-4p_{A})+2p_{a}(1-2p_{A})(1-r)D_{AB}-h_{a}(1-4p_{A}p_{a})\left((1-2p_{A})(1-2p_{B})+2(1-r)D_{AB}\right)\right)$$

$$-(s_{bP}+s_{bO})(1-r)D_{AB}\left(-(1-2p_{A})(1-6p_{B}p_{b})-2(1-2p_{B})(1-r)D_{AB}\right)$$

$+2s_{b}(1-r)D_{AB}\left(p_{b}^{2}(1-2p_{A})(1-4p_{B})+2p_{b}(1-2p_{B})(1-r)D_{AB}-h_{b}(1-4p_{B}p_{b})\left((1-2p_{A})(1-2p_{B})+2(1-r)D_{AB}\right)\right)$. (B7)
