# Supplementary material for: Evolution of Zygotic Linkage Disequilibrium in a Finite Local Population
Source: PLoS One. 2013 Nov 27;8(11):e80538. doi: 10.1371/journal.pone.0080538 (PMC3842346; doi:10.1371/journal.pone.0080538)
Supplement: Appendix S3 — Variances for the per-generation changes in allelic frequency, gametic and zygotic LDs (DOC) [file pone.0080538.s003.doc]

**Appendix S3 Variances for the per-generation changes in allelic frequency, gametic and zygotic LDs**

Variances for the rates of changes in allelic frequency, gametic and zygotic LDs, and other types of covariances, caused by genetic drift in a population of effective size *N*, are derived using Fisher’s delta method [9], [42]. All items with $m_{.}/N$, $s_{.}/N$, and higher orders are neglected in deriving these formulae.

$V(\delta_{p_{A}})=\frac{1}{2N}p_{A}p_{a}$, (C1)

$V(\delta_{p_{B}})=\frac{1}{2N}p_{B}p_{b}$, (C2)

$V(\delta_{D_{AB}})=\frac{1}{2N}\left(p_{A}p_{a}p_{B}p_{b}+(1-2p_{A})(1-2p_{B})(1-r)D_{AB}-(1-r)^{2}D_{AB}^{2}\right)$, (C3)

$$V(\delta_{D_{AABB}})=\frac{1}{N}\left(p_{A}^{2}p_{B}^{2}(1-p_{A}^{2})(1-p_{B}^{2})\right.+2p_{A}p_{B}(1-2p_{A}^{2})(1-2p_{B}^{2})(1-r)D_{AB}$$

$\left.+(1-2p_{A}^{2}-2p_{B}^{2})(1-r)^{2}D_{AB}^{2}-4p_{A}p_{B}(1-r)^{3}D_{AB}^{3}-(1-r)^{4}D_{AB}^{4}\right)$, (C4)

$$V(\delta_{D_{AABb}})=\frac{1}{N}\left(2p_{A}^{2}p_{B}p_{b}(1-\right.p_{A}^{2})(1-2p_{B}p_{b})+2p_{A}(1-2p_{B})(1-4p_{B}p_{b})(1-2p_{A}^{2})(1-r)D_{AB}$$

$\left.-2(1-4p_{B}p_{b})(1-r)^{2}D_{AB}^{2}+8p_{A}(1-2p_{B})(1-r)^{3}D_{AB}^{3}-4(1-r)^{4}D_{AB}^{4}\right)$, (C5)

$$V(\delta_{D_{AaBB}})=\frac{1}{N}\left(2p_{A}p_{a}p_{B}^{2}(1-p_{B}^{2})(1-2p_{A}p_{a})+2p_{B}(1-2p_{A})(1-4p_{A}p_{a})(1-2p_{B}^{2})(1-r)D_{AB}\right.$$

$\left.-2(1-4p_{A}p_{a})(1-r)^{2}D_{AB}^{2}+8p_{B}(1-2p_{A})(1-r)^{3}D_{AB}^{3}-4(1-r)^{4}D_{AB}^{4}\right)$, (C6)

$$V(\delta_{D_{AaBb}})=\frac{1}{N}\left(4p_{A}p_{a}p_{B}p_{b}(1-2p_{A}p_{a})(1-2p_{B}p_{b})+2(1-2p_{A})(1-2p_{B})(1-4p_{A}p_{a})(1-4p_{B}p_{b})(1-r)D_{AB}\right.$$

$\left.-16(1-2p_{A})(1-2p_{B})(1-r)^{3}D_{AB}^{3}-16(1-r)^{4}D_{AB}^{4}\right)$, (C7)

$Cov(\delta_{p_{A}},\delta_{p_{B}})=\frac{1}{2N}(1-r)D_{AB}$, (C8)

$Cov(\delta_{p_{A}},\delta_{D_{AB}})=\frac{1}{2N}(1-2p_{A})(1-r)D_{AB}$, (C9)

$Cov(\delta_{p_{A}},\delta_{D_{AABB}})=\frac{1}{N}\left(p_{A}p_{B}(2-3p_{A})+p_{a}(1-r)D_{AB}\right)(1-r)D_{AB}$, (C10)

$Cov(\delta_{p_{A}},\delta_{D_{AABb}})=\frac{1}{N}\left(p_{A}(1-2p_{B})(2-3p_{A})-2p_{a}(1-r)D_{AB}\right)(1-r)D_{AB}$, (C11)

$Cov(\delta_{p_{A}},\delta_{D_{AaBB}})=\frac{1}{N}\left(p_{B}(1-6p_{A}p_{a})-(1-2p_{A})(1-r)D_{AB}\right)(1-r)D_{AB}$, (C12)

$Cov(\delta p_{A},\delta_{D_{AaBb}})=\frac{1}{N}\left((1-2p_{B})(1-6p_{A}p_{a})+2(1-2p_{A})(1-r)D_{AB}\right)(1-r)D_{AB}$, (C13)

$Cov(\delta_{p_{B}},\delta_{D_{AB}})=\frac{1}{2N}(1-2p_{B})(1-r)D_{AB}$, (C14)

$Cov(\delta_{p_{B}},\delta_{D_{AABB}})=\frac{1}{N}\left(p_{A}p_{B}(2-3p_{B})+p_{b}(1-r)D_{AB}\right)(1-r)D_{AB}$, (C15)

$Cov(\delta_{p_{B}},\delta_{D_{AABb}})=\frac{1}{N}\left(p_{A}(1-6p_{B}p_{b})-(1-2p_{B})(1-r)D_{AB}\right)(1-r)D_{AB}$, (C16)

$Cov(\delta_{p_{B}},\delta_{D_{AaBB}})=\frac{1}{N}\left(p_{B}(1-2p_{A})(2-3p_{B})-2p_{b}(1-r)D_{AB}\right)(1-r)D_{AB}$, (C17)

$Cov(\delta_{p_{B}},\delta_{D_{AaBb}})=\frac{1}{N}\left((1-2p_{A})(1-6p_{B}p_{b})+2(1-2p_{B})(1-r)D_{AB}\right)(1-r)D_{AB}$, (C18)

$$Cov(\delta_{D_{AB}},\delta_{D_{AABB}})=\frac{1}{N}\left(p_{A}^{2}p_{B}^{2}p_{a}p_{b}+p_{A}p_{B}\left(p_{a}p_{b}+(1-2p_{A})(1-2p_{B})\right)(1-r)D_{AB}\right.$$

$\left.+(p_{a}p_{b}-2p_{A}p_{B})(1-r)^{2}D_{AB}^{2}-(1-r)^{3}D_{AB}^{3}\right)$, (C19)

$$Cov(\delta_{D_{AB}},\delta_{D_{AABb}})=\frac{1}{N}\left(p_{A}^{2}p_{a}p_{B}p_{b}(1-2p_{B})+p_{A}\left(-2p_{B}p_{b}(2-3p_{A})+(1-2p_{A})(1-2p_{B}p_{b})\right)\right.(1-r)D_{AB}$$

$\left.-(1-2p_{B})(1+p_{A})(1-r)^{2}D_{AB}^{2}+2(1-r)^{3}D_{AB}^{3}\right)$, (C20)

$$Cov(\delta_{D_{AB}},\delta_{D_{AaBB}})=\frac{1}{N}\left(p_{A}p_{a}p_{B}^{2}p_{b}(1-2p_{A})+p_{B}\left((1-2p_{B})(1-2p_{A}p_{a})-2p_{A}p_{a}(2-3p_{B})\right)(1-r)D_{AB}\right.$$

$\left.-(1-2p_{A})(1+p_{B})(1-r)^{2}D_{AB}^{2}+2(1-r)^{3}D_{AB}^{3}\right)$, (C21)

$$Cov(\delta_{D_{AB}},\delta_{D_{AaBb}})=\frac{1}{N}\left(p_{A}p_{a}p_{B}p_{b}(1-2p_{A})(1-2p_{B})+\left((1-2p_{A})^{2}(1-2p_{B})^{2}+4p_{A}p_{a}p_{B}p_{b}\right)\right.(1-r)D_{AB}$$

$\left.-(1-2p_{A})(1-2p_{B})(1-r)^{2}D_{AB}^{2}-4(1-r)^{3}D_{AB}^{3}\right)$, (C22)

$$Cov(\delta_{D_{AABB}},\delta_{D_{AABb}})=\frac{1}{N}\left(-2p_{A}^{2}p_{B}^{3}p_{b}(1-p_{A}^{2})+2p_{A}p_{B}^{2}(p_{B}-3p_{b})(1-2p_{A}^{2})(1-r)D_{AB}\right.$$

$\left.-2p_{B}(1-2p_{B})(1-r)^{2}D_{AB}^{2}-2p_{A}(p_{b}-3p_{B})(1-r)^{3}D_{AB}^{3}+2(1-r)^{4}D_{AB}^{4}\right)$, (C23)

$$Cov(\delta_{D_{AABB}},\delta_{D_{AaBB}})=\frac{1}{N}\left(-2p_{A}^{3}p_{a}p_{B}^{2}(1-p_{B}^{2})+2p_{A}^{2}p_{B}(1-2p_{B}^{2})(p_{A}-3p_{a})(1-r)D_{AB}\right.$$

$\left.-2p_{A}(1-2p_{A})(1-r)^{2}D_{AB}^{2}-2p_{B}(p_{a}-3p_{A})(1-r)^{3}D_{AB}^{3}+2(1-r)^{4}D_{AB}^{4}\right)$, (C24)

$$Cov(\delta_{D_{AABB}},\delta_{D_{AaBb}})=\frac{1}{N}\left(4p_{A}^{3}p_{a}p_{B}^{3}p_{b}+2p_{A}^{2}p_{B}^{2}\left((1-4p_{A})(1-4p_{B})+8(1-p_{A}-p_{B})\right)(1-r)D_{AB}\right.$$

$\left.-2\left((1-2p_{A})(1-2p_{B})+4p_{A}p_{B}\right)(1-r)^{3}D_{AB}^{3}-4(1-r)^{4}D_{AB}^{4}\right)$, (C25)

$$Cov(\delta_{D_{AABb}},\delta_{D_{AaBB}})=\frac{1}{N}\left(4p_{A}^{3}p_{a}p_{B}^{3}p_{b}+2p_{A}^{2}p_{B}^{2}(3-4p_{B})(3-4p_{A})(1-r)D_{AB}\right.$$

$\left.+4(p_{A}+p_{B}-4p_{A}p_{B})(1-r)^{3}D_{AB}^{3}-4(1-r)^{4}D_{AB}^{4}\right)$, (C26)

$$Cov(\delta_{D_{AABb}},\delta_{D_{AaBb}})=\frac{1}{N}\left(-4p_{A}^{3}p_{a}p_{B}p_{b}(1-2p_{B}p_{b})-2p_{A}^{2}(1-2p_{B})(1-4p_{B}p_{b})(3-4p_{A})(1-r)D_{AB}\right.$$

$\left.+4(1-2p_{B})(1-4p_{A})(1-r)^{3}D_{AB}^{3}+8(1-r)^{4}D_{AB}^{4}\right)$, (C27)

and

$$Cov(\delta_{D_{AaBB}},\delta_{D_{AaBb}})=\frac{1}{N}\left(-4p_{A}p_{a}p_{B}^{3}p_{b}(1-2p_{A}p_{a})-2p_{B}^{2}(1-2p_{A})(1-4p_{A}p_{a})(3-4p_{B})(1-r)D_{AB}\right.$$

$\left.+4(1-2p_{A})(1-4p_{B})(1-r)^{3}D_{AB}^{3}+8(1-r)^{4}D_{AB}^{4}\right)$. (C28)
