# Supplementary material for: Evolution of Zygotic Linkage Disequilibrium in a Finite Local Population
Source: PLoS One. 2013 Nov 27;8(11):e80538. doi: 10.1371/journal.pone.0080538 (PMC3842346; doi:10.1371/journal.pone.0080538)
Supplement: Appendix S4 — Expectations of variances of zygotic LDs and the covariances between gametic and zygotic LDs (DOC) [file pone.0080538.s004.doc]

**Appendix S4 Expectations of the variances of zygotic LDs and the covariances between gametic and zygotic LDs**

First, prove the equality$E(V(D_{....}))=E(V(\delta_{D_{....}}))$where *D….* refers to one of the four independent zygotic LDs (*D_AABB_*, *D_AaBB_*, *D_AABb_*, and *D_AaBb_*). Let $D_{....}^{**}$ be the zygotic LD before the occurrence of genetic drift but after selection in the sporophyte stage, and $\Delta D_{....}$=$D_{....}^{**}-D_{....}$ be the difference of $D_{....}^{**}$from its value in the preceding adults.$\Delta D_{....}$ is the deterministic change due to the effects of migration and selection before genetic drift. After genetic drift, let $D'_{....}$be the zygotic LD, which can be expressed as where $\delta'_{D_{....}}$be the random change due to the genetic drift effects. Let $\delta_{D_{....}}=D'_{....}-D_{....}$, the difference of zygotic LD per generation, and then $\delta_{D_{....}}=D_{....}^{**}+\delta'_{D_{....}}-D_{....}$=$\Delta D_{....}+\delta'_{D_{....}}$. Note that the deterministic component $\Delta D_{....}$is independent of the random change caused by the genetic drift effects,$\delta'_{D_{....}}$. From the above settings, I can obtain

$V(\delta_{D_{....}})=E_{\delta}(\delta_{D_{....}}^{2})-(E_{\delta}(\delta_{D_{....}}))^{2}$, (D1)

where $E_{\delta}$is the operator of taking the expectation with respect to the change $\delta_{D_{....}}$[12].

Equation (D1) can be expanded in the following way:

=

=

=

=$V(D'_{....})$. (D2)

$V(D'_{....})$is the variance of zygotic LD caused by genetic drift (similar to sampling), and can be approximated using Fisher’s delta method ([9], p.49). Thus, the expectation of steady-state $V(\delta_{D_{....}})$ can be approximated by the expectation of steady-state $V(D_{....})$, i.e. $E(V(D_{....}))=E(V(\delta_{D_{....}}))$where *E* is the operator of taking the expectation with respect to the steady-state density distribution function$\Phi$.

Next, prove$E(\mathrm{cov}(D_{AB},D_{....}))=E(\mathrm{cov}(\delta_{D_{AB},}\delta_{D_{....}}))$. Similarly, let $D_{AB}^{**}$ be the gametic LD before the occurrence of genetic drift but after selection in the sporophyte stage, and $\Delta D_{AB}$=$D_{AB}^{**}-D_{AB}$ be the deterministic change due to the effects of migration and selection. Let $D'_{AB}$be the zygotic LD after genetic drift and in which $\delta'_{D_{AB}}$be the random change due to the genetic drift effects. Let $\delta_{D_{AB}}=D'_{AB}-D_{AB}$ the difference per generation, and then $\delta_{D_{AB}}=D_{AB}^{**}+\delta'_{D_{AB}}-D_{AB}$=$\Delta D_{AB}+\delta'_{D_{AB}}$.

From the above settings, I can obtain

$$\mathrm{cov}(\delta_{D_{AB}},\delta_{D_{....}})=E_{\delta}(\delta_{D_{AB}}\delta_{D_{....}})-E_{\delta}(\delta_{D_{AB}})E_{\delta}(\delta_{D_{....}})$$

=

=

=

=

=$\mathrm{cov}(D'_{AB},D'$. (D3)

$Cov(D'_{AB},D'$is the covariance between gametic and zygotic LDs caused by genetic drift, and can be directly calculated using Fisher’s delta method ([9], p.118). The expectation of steady-state $\mathrm{cov}(\delta_{D_{AB}},\delta_{D_{....}})$can be approximated by the expectation of steady-state$\mathrm{cov}(D_{AB},D_{....})$, i.e. $E(\mathrm{cov}(D_{AB},D_{....}))=E(\mathrm{cov}(\delta_{D_{AB},}\delta_{D_{....}}))$.
